# Safety and Immunogenicity of Malaria Vectored Vaccines Given with Routine Expanded Program on Immunization Vaccines in Gambian Infants and Neonates: A Randomized Controlled Trial

**DOI:** 10.3389/fimmu.2017.01551

**Published:** 2017-11-20

**Authors:** Victorine A. Mensah, Sophie Roetynck, Ebrima K. Kanteh, Georgina Bowyer, Amy Ndaw, Francis Oko, Carly M. Bliss, Ya Jankey Jagne, Riccardo Cortese, Alfredo Nicosia, Rachel Roberts, Flavia D’Alessio, Odile Leroy, Babacar Faye, Beate Kampmann, Badara Cisse, Kalifa Bojang, Stephen Gerry, Nicola K. Viebig, Alison M. Lawrie, Ed Clarke, Egeruan B. Imoukhuede, Katie J. Ewer, Adrian V. S. Hill, Muhammed O. Afolabi

**Affiliations:** ^1^Université Cheikh Anta Diop, Dakar, Senegal; ^2^Medical Research Council Unit, Fajara, Gambia; ^3^The Jenner Institute Laboratories, University of Oxford, Oxford, United Kingdom; ^4^Keires AG, Basel, Switzerland; ^5^ReiThera, Rome, Italy; ^6^CEINGE, Naples, Italy; ^7^Department of Molecular Medicine and Medical Biotechnology, University Federico II, Naples, Italy; ^8^Centre for Clinical Vaccinology and Tropical Medicine, The Jenner Institute, Churchill Hospital, Oxford, United Kingdom; ^9^European Vaccine Initiative, UniversitätsKlinikum Heidelberg, Heidelberg, Germany; ^10^Centre for International Child Health, Imperial College London, London, United Kingdom; ^11^Centre for Statistics in Medicine, Botnar Research Centre, Nuffield Department of Orthopaedics, Rheumatology and Musculoskeletal Sciences, University of Oxford, Oxford, United Kingdom

**Keywords:** vaccines, clinical trials, malaria, cellular immune response, cytokines

## Abstract

**Background:**

Heterologous prime-boost vaccination with chimpanzee adenovirus 63 (ChAd63) and modified vaccinia virus Ankara (MVA) encoding multiple epitope string thrombospondin-related adhesion protein (ME-TRAP) has shown acceptable safety and promising immunogenicity in African adult and pediatric populations. If licensed, this vaccine could be given to infants receiving routine childhood immunizations. We therefore evaluated responses to ChAd63 MVA ME-TRAP when co-administered with routine Expanded Program on Immunization (EPI) vaccines.

**Methods:**

We enrolled 65 Gambian infants and neonates, aged 16, 8, or 1 week at first vaccination and randomized them to receive either ME-TRAP and EPI vaccines or EPI vaccines only. Safety was assessed by the description of vaccine-related adverse events (AEs). Immunogenicity was evaluated using IFNγ enzyme-linked immunospot, whole-blood flow cytometry, and anti-TRAP IgG ELISA. Serology was performed to confirm all infants achieved protective titers to EPI vaccines.

**Results:**

The vaccines were well tolerated in all age groups with no vaccine-related serious AEs. High-level TRAP-specific IgG and T cell responses were generated after boosting with MVA. CD8^+^ T cell responses, previously found to correlate with protection, were induced in all groups. Antibody responses to EPI vaccines were not altered significantly.

**Conclusion:**

Malaria vectored prime-boost vaccines co-administered with routine childhood immunizations were well tolerated. Potent humoral and cellular immunity induced by ChAd63 MVA ME-TRAP did not reduce the immunogenicity of co-administered EPI vaccines, supporting further evaluation of this regimen in infant populations.

**Clinical Trial Registration:**

The clinical trial was registered on http://Clinicaltrials.gov (NCT02083887) and the Pan-African Clinical Trials Registry (PACTR201402000749217).

## Introduction

Recent estimates from the World Health Organization (WHO) show that many African countries are adopting preventive measures toward malaria elimination (1). However, achieving this goal would need new interventions to complement current control strategies. To curtail the threat posed by emerging resistance to artemisinin-based chemotherapy and insecticide-treated bed nets, efforts have intensified to develop potent vaccines as a complementary tool (2, 3).

The most clinically advanced malaria vaccine to date, RTS,S, has limited efficacy coupled with some safety concerns and practical deployment challenges for its four dose regimen in target age groups at high risk of malaria (4). Novel approaches to generate immunity to malaria employing viral vector vaccines were initially limited due to the use of homologous prime-boost approaches that induced relatively poor T cell responses (5, 6). These have been improved by the development of a novel heterologous approach, which elicits potent and durable cell-mediated immunity (7).

Since 2007, we have extensively evaluated heterologous prime-boost vaccination with chimpanzee adenovirus 63 (ChAd63) and modified vaccinia virus Ankara (MVA) expressing the *Plasmodium falciparum* pre-erythrocytic antigen ME-TRAP, a multiple epitope string (ME) fused to thrombospondin-related adhesion protein (TRAP) among malaria-naïve adults in Europe and malaria-exposed adults, children, and infants in Africa (8–11). High-level efficacy was observed in Kenyan adults (66%) over 8 weeks of follow-up (12). Across four de-escalating pediatric age groups in Burkina Faso and The Gambia, these vaccines had acceptable safety profiles and induced primarily CD8^+^ T cells and potent humoral immunity (13, 14).

Due to the very high malaria burden among young infants in sub-Saharan Africa, a malaria vaccine should be given in early infancy to provide effective protection. During this period, routine vaccines are administered to infants according to the WHO Expanded Program on Immunization (EPI) schedule. Incorporating a new malaria vaccine within an existing EPI schedule would facilitate acceptance and uptake by parents and reduce logistical costs. However, coadministration with EPI vaccines can reduce immunogenicity of new candidate vaccines (15); hence, it is crucial to evaluate early the immune responses of co-administered vaccines. Such evaluation could also guide the selection of optimal vaccination schedules and appropriate infant age groups for large efficacy trials (16). Coadministration of RTS,S with diphtheria, tetanus, and pertussis (DTP)-containing vaccines in a late phase trial may have contributed to the low immune responses observed among infants aged 6–12 weeks (17). Similarly, coadministration of a candidate tuberculosis vaccine, MVA85A, with EPI vaccines resulted in a drop in tuberculosis-specific immunogenicity (15). These findings underscore the importance of appropriate timing of immunizations, both to inform the design of efficacy trials involving T cell-inducing vaccines and also to prioritize identification of optimal schedules for integration within the EPI program (16).

In this study, we investigated the safety and immunogenicity of ChAd63 MVA ME-TRAP when co-administered with EPI vaccines among Gambian infants aged 16, 8, and 1 week at first immunization.

## Materials and Methods

### Objectives

The primary objective was to evaluate the safety and reactogenicity of ChAd63 ME-TRAP and MVA ME-TRAP prime-boost immunization when co-administered with EPI vaccines in healthy Gambians at ages of 1 and 8, 8 and 16, or 16 and 24 weeks, respectively. Secondary objectives were to evaluate both the cellular and humoral immunogenicity of ChAd63 MVA ME-TRAP and the antibody responses to EPI vaccines when given alone to unvaccinated controls or when co-administered with the candidate vaccines within the standard Gambian EPI schedule.

### Study Setting

The trial was undertaken from May 2014 to November 2015 at the Sukuta field site of Medical Research Council Unit The Gambia (MRCG). Sukuta is a peri-urban Gambian settlement located about 25 km south of the capital, Banjul. Malaria transmission is highly seasonal in this setting, occurring almost exclusively from July to January, with a peak in November (18). The predominant malaria vector is *Anopheles gambiae*.

The MRCG has a 42-bed facility for in-patient care. This is supported by laboratory and radiology diagnostic facilities. Children including neonates presenting with fever (>37.5°C) or hypothermia (<35.5°C) lasting more than 24 h in addition to danger signs such as convulsion, refusal of feeds, and abnormal cry would undergo full septic work-up. These include comprehensive clinical evaluation to identify the source of infection. To establish the definitive diagnosis, investigations such as full blood count, blood culture and CSF analysis are done. Antibiotic management is shaped by the result of these tests. Supportive management including fluid management is provided based on the clinical needs of the neonates.

### Participants

Sensitization meetings were held with the local community to identify potential participants for the 16- and 8-week cohorts. Prior to enrollment, mothers gave written informed consent after the trial information had been explained clearly. For the 1-week-old group, pregnant women attending antenatal care at the government health center located within the field site were sensitized from 30 to 32 weeks of gestation. The trial team attended the delivery of consenting pregnant women and conducted an eligibility assessment and screening tests on the newborn infants.

Exclusion criteria included any evidence of acute or chronic illness or of hematological, renal or hepatic pathology, birth weight less than 2.5 kg, significant antenatal, perinatal or early postnatal complications, weight for age *z*-scores below 2 SDs of normal for age, hemoglobin less than 10 g/dl at >4 weeks of age or less than 13.0 g/dl at <4 weeks of age, white cell count <5.0 × 10^9^/l, positive malaria antigen test, positive maternal HIV serology test, clinically significant serum biochemistry results, prior receipt of an investigational malaria vaccine, recent or planned use of any investigational drug, vaccine, immunoglobulin or any blood product, use of immunosuppressant drugs, confirmed or suspected immunodeficiency, history of surgical splenectomy, and concurrent participation in another clinical trial. The infants must have received primary EPI vaccines according to defined schedules.

### Study Design

**Figure 1:**
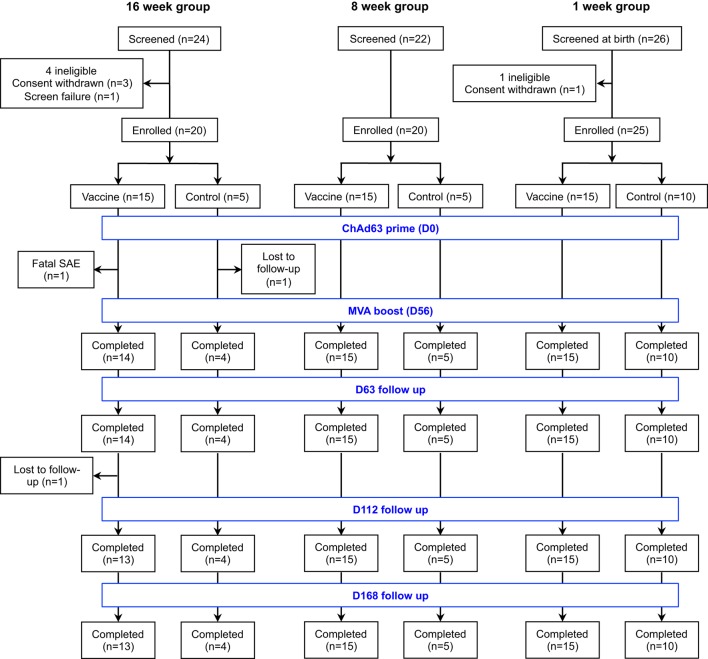
Flow of study design and volunteer enrollment. Seventy-two infants and neonates were screened for eligibility across the three age groups and 65 eligible infants were enrolled, randomized, vaccinated according to randomization list and followed up for 252 days. Of 24 infants screened in the 16-week-old group, mothers of 3 infants withdrew consent before enrollment while 1 infant was excluded due to markedly raised alanine transaminase. Among those randomized to the vaccine arm, one infant had fatal SAE that was non-related to the study vaccines and another was lost to follow-up due to relocation of mother, while only one infant did not complete the follow-up in the control arm. Twenty-two infants were screened for the 8-week-old cohort and all were eligible. Only the first 20 were randomized into 15 vaccine group and 5 controls. Similarly, 26 mother-newborn pairs were screened for the 1-week-old group. A mother withdrew consent before enrollment and the remaining 25 neonates were randomized into 15 vaccinees and 10 controls. All study infants in the 8- and 1-week-old groups completed the study follow-up. SAE, serious adverse event.

The trial comprised three groups enrolled in an age de-escalation pattern (Figure 1). The first and second groups had 5 infants randomized to receive only EPI vaccines while 10 neonates were assigned to EPI vaccines only (control) arm in the third group because considerable rates of concurrent illnesses were anticipated in the neonatal age group.

The candidate vaccines were administered intramuscularly in the three age groups in the left antero-lateral thigh, while the EPI vaccines were administered on the right antero-lateral thigh to ensure objective comparison of reactogenicity and solicited AEs across the study arms. All infants randomized to the vaccine arm received ChAd63 ME-TRAP (5 × 10^10^ vp) at 1, 8, or 16 weeks of age accordingly, followed 8 weeks later by MVA ME-TRAP (1 × 10^8^ pfu). All infants were given EPI vaccination according to the schedule implemented in the Gambia as follow: BCG and first dose of oral polio vaccine (OPV) and hepatitis B vaccine within 2 weeks of birth, doses 2, 3, and 4 of OPV, and 3 dose primary series of 13-valent pneumococcal conjugate, rotavirus (Rotateq), and DTP *Haemophilus influenza* type B (Hib), Hepatitis B pentavalent vaccine at 8, 12, and 16 weeks, followed by measles and yellow fever vaccine at 36 weeks of age (Table S1 in Supplementary Material).

### Randomization and Blinding

An independent statistician at the Centre for Statistics in Medicine, University of Oxford, performed a block randomization of eligible infants for each age group. Sealed envelopes labeled with a unique code containing the treatment allocated to each eligible infant were provided according to the randomization list. This guaranteed treatment concealment until enrollment as the envelopes were opened only after the study infants were enrolled. However, the clinical investigators and mothers of study infants were not blinded to vaccination regimen but the laboratory staff who conducted the EPI antibody testing were blinded to the study arms.

### Sample Size

As this was a Phase Ib trial, the sample size was shaped by the need to balance the number required for evaluation of safety and immunogenicity of an investigational vaccine with the risk of exposing a large group of study participants to an unlicensed vaccine. Thus, the sample size was not powered to detect differences between the study arms.

### Interventions

Details about the study vaccines have been described elsewhere (8–10). Briefly, ChAd63 ME-TRAP and MVA ME-TRAP were respectively manufactured by Clinical Biomanufacturing Facility, University of Oxford, UK, and Impfstoffwerke Dessau-Tornau, Germany, under Good Manufacturing Practice conditions.

### Outcomes

The primary endpoint was safety measured as (i) occurrence of solicited symptoms during a 3-day follow-up period after each immunization; (ii) occurrence of unsolicited symptoms during a 30-day follow-up after each vaccination; (iii) occurrence of abnormal laboratory results during study period; and (iv) occurrence of serious AEs throughout the study period.

### Assessment of Primary Endpoints (Safety and Reactogenicity)

After each vaccination, all study infants and neonates were directly observed in the trial clinic for 1 h and followed up for occurrence of solicited symptoms for three consecutive days. The study participants were further assessed for unsolicited symptoms for 30 days after each study vaccination. Laboratory abnormalities and SAEs were assessed for the entire study period (Table 4). Trained field assistants visited the infants at home daily for the following 3 days after each vaccination to administer a standardized reactogenicity card to the mothers, which included history of fever, vomiting, diarrhea, reduced oral intake, and reduced activities. The field assistants also examined the infants for expected local AEs (swelling, tenderness, limitation of arm movement, redness and desquamation at the site of injection) and fever. Pain at the injection site was graded on a scale of 0–3 where 0 = no pain, 1 = painful to touch, 2 = pain when arm is touched, and 3 = severe pain at rest.

Further safety assessments were subsequently performed on all study infants on days 7, 21, 63, 112, 168, 224, and 252 after enrollment. Clinical evaluations consisted of measurement of vital signs and assessment for local injection site and general solicited symptoms and signs. Local solicited symptoms and signs included pain, swelling, redness at injection site while systemic solicited symptoms and signs included fever (axillary temperature of >38.0°C), reduced oral intake, reduced activity, and vomiting. Any other symptoms or signs were considered unsolicited and were recorded during the 30 days after each vaccination while SAEs were monitored throughout the study period. Blood samples were collected at screening visit and study days 21, 63, 112, and 168 to determine complete blood count, alanine transaminase (ALT), and serum creatinine.

Adverse events were graded by intensity and judged for relatedness to study vaccines. Mild AEs were easily tolerated, causing minimal discomfort and not interfering with daily activities. Moderate AEs were sufficiently discomforting to interfere with normal activities. Severe AEs prevented normal daily activities. Swelling, redness and fever had specific definitions not based on interference with daily activities. Injection site swelling and redness were graded based on their widest dimension: mild, 0–20 mm; moderate, 20–50 mm; and severe, >50 mm. Fever was classified as severe if the axillary temperature was ≥40.0°C. For laboratory tests, toxicity grading was adapted to normal reference ranges determined for the local infant population (19).

### Blood Processing

Blood samples were collected using the heparinized Vacutainer^®^ system (Becton Dickinson) and stored at room temperature (RT) prior to processing, which was completed within 6 h of venipuncture. PBMC were separated by density centrifugation from whole blood and re-suspended in RPMI (Sigma) containing 10% heat-inactivated, batch tested, sterile-filtered fetal calf serum (FCS) previously screened for low reactivity (Labtech International), 1% l-glutamine, and 1% penicillin/streptomycin. Cell counts were performed using trypan blue (Sigma) staining and a microscope according to an established laboratory SOP.

### *Ex Vivo* Enzyme-Linked Immunospot (ELISpot) Assays

*Ex vivo* (18-h stimulation) ELISpot assays were performed using Multiscreen IP ELISpot plates (Millipore), human IFNγ SA-ALP antibody kits (Mabtech), and BCIP NBT-plus chromogenic substrate (Moss Inc). Cells were cultured in RPMI containing 10% heat-inactivated, sterile-filtered FCS, supplemented with 1% l-glutamine, and 1% penicillin/streptomycin. Antigens were tested in duplicate with 250,000 PBMC added to each well of the ELISpot plate. TRAP peptides were 20 amino acids in length, overlapping by 10 amino acids (NeoBioLab), assayed in 6 pools of 7–10 peptides at a final concentration of 10 µg/ml. Responses were averaged across duplicates and responses in unstimulated (negative control) wells were subtracted. Responses to the T9/96 strain of the TRAP antigen were evaluated by summing responses to individual pools. Responses to TRAP from 3D7 strain were tested using a single pool of peptides covering the entire sequence of the TRAP antigen. Staphylococcal Enterotoxin B (0.02 µg/ml) and phytohemmagglutinin-L (10 µg/ml) were used as positive control. Plates were counted using an AID automated ELISpot counter (AID Diagnostika GmbH, algorithm C) and using identical settings for all plates, and counts were adjusted only to remove artifacts. Responses to the negative control were always less than 20 spot-forming cells (SFC) per well.

Pools were considered positive if the response was both greater than the background response plus five SFC and two times higher than the negative control for that assay. The lower limit of detection (LLD) for the assay was 4 SFC/million PBMC for individual pairs of wells and 28 SFC/million PBMC for the response to ME-TRAP.

### Whole Blood Stimulation for Intracellular Cytokine Staining (ICS) Analysis

Thrombospondin-related adhesion protein-specific CD4^+^ and CD8^+^ T cell responses were further characterized in blood samples taken from vaccinees, 21 days post-priming with ChAd63 ME-TRAP and 7 days post-boosting with MVA ME-TRAP (at day 63) by intracellular staining. Secretion of IFNγ, IL-2, and TNFα by T cells was measured following *in vitro* recall with a single pool of peptides spanning the entire sequence of TRAP protein from T9/96 *P. falciparum* strain.

The whole blood ICS protocol was adapted from Hanekom et al. (20) and optimized to include dead cell identification through non-fixation of samples ahead of red cell lysis, thus enabling a cell viability readout, in addition to the exclusion of dead or dying cells, which often auto-fluoresce and/or non-specifically bind the antibodies used in flow cytometry. 350 µl of blood were distributed in 2 ml screw-cap tubes (Sarstedt) and stimulated in the presence of 0.5 µg/ml of anti-CD49d and anti-CD28 monoclonal antibodies (eBioscience) and a final concentration of 2 µg/ml of a pool of 56 20mers, overlapping by 10 amino acids, covering the full sequence of the TRAP antigen from the T9/96 *P. falciparum* strain (Neopeptide). Staphylococcal Enterotoxin B (Sigma) at 2 µg/ml or an equivalent volume of RPMI medium supplemented with 10% heat-inactivated FCS, 1% penicillin/streptomycin, and 1% l-glutamine were used as positive and negative controls, respectively. Tubes were incubated at 37°C in 5% CO_2_ for 2–4 h, prior to the addition of 1 µg/ml Brefeldin A (eBioscience) and further incubation at 37°C for 16 h. At the end of the culture, samples were then incubated with 2 mM EDTA (Gibco) for 15 min to remove adherent cells, then treated for 2 × 10 min with Red Cell Lysis Buffer (Qiagen). Cells were then stained at RT for 20 min in the dark with Live/Dead fixable Aqua amine reactive dye (Life Technologies) for live cell identification and fixed with 1% paraformaldehyde (Sigma) in phosphate-buffered saline (PBS) for 5 min. Cells were subsequently frozen in 0.5 ml FCS containing 10% DMSO (Sigma) and stored at −80°C until further use for batched analysis.

### Multiparameter Flow Cytometry Analysis

The T cell cytokine response to the pooled peptides was assessed on stimulated whole blood using an 8-color antibody panel. Cells were thawed and stained in batches within 4 months of the date they were frozen. Each participant’s day 21 and day 63 samples were analyzed on the same day for consistency. After thawing, cells were transferred in polystyrene FACS tubes and washed with FACS buffer [PBS containing 0.1% bovine serum albumin (BSA) and 0.01% sodium azide (both from Sigma)]. Samples were then prepared following a staining protocol standardized across all trial sites (11, 14). After 20 min of permeabilization in Cytofix/Cytoperm buffer (BD Biosciences) at RT in the dark, cells were washed once in 1× Perm/Wash Buffer (BD Biosciences). Cells were then stained at RT for 30 min with a combined surface and intracellular cocktail containing CD3 AlexaFluor700 (1/50, clone UCHT1), CD4 APC (1/25, clone Leu-3), CD8 APC-eFluor780 (1/10, clone RPAT8), CD14 eFluor450 (1/50, clone 61D3), CD19 eFluor450 (1/50, clone SJ25-C1), IFNγ FITC (1/100, clone 4S.B3), IL-2 PE (1/50, clone MQ1-17H12), and TNFα PECy7 (1/500, clone MAb11) monoclonal antibodies (all from eBioscience). Samples were washed in 1× Perm/Wash Buffer and re-suspended in 1% paraformaldehyde (Sigma) in PBS for consistency across all trial sites due to logistic issues that can arise at acquisition. Samples were then analyzed using a BD LSR Fortessa Cell Analyzer (Becton Dickinson). Compensation was calculated for each fluorochrome on each acquisition run using OneComp beads (eBioscience) for surface and intracellular antibodies and ARC beads (Life Technologies) for Aqua reactive dye. An average of 700,000 total events (IQR: 493,669–851,834 events) was collected per sample. Figure S1 in Supplementary Material shows the hierarchical gating strategy for this analysis. A minimum of 55,000 live CD3^+^ lymphocytes was analyzed for each sample. At least 23,000 CD4^+^ or CD8^+^ T cells were analyzed for multiple cytokine expression after exclusion of any double positive cells, respectively. Any sample with less than 23,000 events in the CD4^+^ or CD8^+^ gate was excluded. 8 out of 88 samples (corresponding to 1 sample for the CD4^+^ and 7 for the CD8^+^ T cell cytokine response analysis) with a total cytokine response to the positive control lower than 1% cytokine positive CD4^+^ or CD8^+^ T cells were further excluded from subsequent analysis. Responses were calculated after background subtraction, corresponding to the response in the medium control for each sample. The LLD of the assay, corresponding to 1/minimum number of events in the CD4^+^ or CD8^+^ gate*100, was 0.0047% of the parent population for all three cytokines for both CD4^+^ and CD8^+^ T cells. A positive response was strictly greater than one time the unstimulated control of the corresponding sample. Any response failing this criterion was considered negative and was replaced by LLD value or excluded from analysis when stated otherwise. Bar charts were created using absolute frequencies. Pie charts were generated using relative frequencies with a threshold of 0.004% of the parent population. Data analysis was performed using FlowJo v10.1r1 (Treestar Inc., USA), Pestle v1.7, and Spice v5.3 (Mario Roederer, Vaccine Research Centre, NIAID, NIH, USA).

### TRAP-Specific Total IgG ELISA

Antibody responses were measured by anti-TRAP IgG sandwich ELISA. Nunc-Immuno 96 well plates were coated with 0.5 µg/ml of TRAP antigen from 3D7 *Pf* strain in carbonate-bicarbonate coating buffer and left overnight at 4°C. Plates were washed six times with phosphate-buffered saline-Tween (PBS/T), then blocked with 1% BSA in PBS/T for 1 h at RT. Serum was diluted in PBS/T containing 0.2% BSA at concentrations of 1:100, 1:500, or 1:1,000, and added in triplicate. Serum samples from the screening visit, days 21, 56, 63, 112, and 168, were analyzed. Plates were incubated at RT for 2 h then washed as before. A secondary antibody (goat anti-human whole IgG conjugated to alkaline phosphatase, Sigma) was added at a dilution of 1:1,000 in PBS/T 0.2% BSA for 1 h at RT. After a final wash, plates were developed by adding 4-nitrophenyl phosphate in diethanolamine buffer (Pierce). A positive reference standard (made from pooled TRAP-positive serum) was used on each plate to give a standard curve. It was added in duplicate at an initial dilution of 1:100 (in PBS/T 0.2% BSA) and diluted twofold 10 times, starting with an arbitrary value of 20 antibody units. Four blank wells (0 antibody units) were also designated. The optical density (OD) values were then fitted to a four-parameter standard curve using SOFTmax PRO software. An internal control was included on every plate in triplicate made up from a 1:800 dilution (in PBS/T 0.2% BSA) of the positive standard. OD was read at 405 nm using an ELx800 microplate reader. Test sera antibody units were calculated from their OD values using the parameters estimated from the standard curve. Previously published data shown for comparison in Figure 7B was generated using the same ELISA assay performed in the same laboratory by the same technician and is therefore comparable to this dataset.

### Serology to EPI Vaccines

Serology for responses to EPI vaccines at 24 weeks (8 weeks after last primary EPI) was performed at the National Institute for Public Health and the Environment (RIVM) in the Netherlands. IgG antibodies directed against *Bordetella pertussis*, diphtheria, tetanus, Hib, and 13 *Streptococcus pneumoniae* serotypes were measured in duplicate using fluorescent bead-based multiplex immuno assays (Luminex xMAP technology) (21–23). In all assays, a reference, controls and blanks were included on each plate. All analyses were performed with a Bio-Plex 200 in combination with Bio-Plex manager software (Bio-Rad Laboratories, Hercules, CA, USA). For the *B. pertussis*, diphtheria and tetanus (DTaP) multiplex assay, samples were diluted 1:200 and 1:4,000 in PBS containing 0.1% Tween-20 and 3% BSA. Serum values for *B. pertussis* were assigned in endotoxin units per milliliter as the used in-house reference was calibrated against the U.S. Reference Pertussis Anti-serum Human lot 3 (CBER/FDA). Hib IgG antibodies were determined similar to the DTaP multiplex assay, with the exception that sera were diluted 1:100 and 1:400 in 50% antibody depleted human serum. Values were expressed in micrograms per milliliter as the used in-house reference was calibrated against lot 1983 (CBER/FDA). Pneumococcal IgG antibody concentrations of serotypes included in PCV13 were simultaneously measured by diluting the sera 1:1,000 in PBS containing 15 µg/ml cell wall polysaccharide Multi (Statens Serum Institute, Copenhagen, Denmark) and 5% antibody depleted human serum to reduce non-specific reactions. Serum values were expressed in µg/ml as the used in-house reference was calibrated against lot 89-S serum (CBER/FDA). For Hepatitis B, antibodies to the surface antigen were measured using the Abbot Architect 2000i chemiluminescent micro-particle immunoassay, product # 7C18. Antibody concentration is determined against a calibration curve with a concentration of 10.0 mIU/ml or greater considered positive.

### Statistical Methods

Safety and clinical laboratory data were double-entered on OpenClinica^®^ software and analyses were performed using STATA Release statistical software version 14.1 (StataCorp LP, College Station, TX, USA). For categorical variables, data were summarized using absolute numbers and percentages and groups were compared with Fisher’s exact test (FET). For continuous variables, the median and inter-quartile range or geometric mean with 95% confidence interval (CI) were used to summarize the data. Intent to treat analysis was used. For immunological data, statistical analyses were performed using GraphPad Prism, Mac version 6 (GraphPad Software Inc., USA) or Spice v5.3 (Mario Roederer, Vaccine Research Centre, NIAID, NIH, USA). Geometric mean responses are shown for each group, unless stated otherwise in the figure legends. Differences in ELISpot responses over the time course of follow-up were tested for significance using the Friedman matched-pairs non-parametric test, while variations in responses between individual time points were compared for each infant within each age group using the Wilcoxon matched-pairs signed rank test, respectively. Infants with missing data at any time point were excluded from matched-pairs analyses. Mann–Whitney *U* test was used to compare responses in vaccinees to those of controls within the same age group at a specific time point. Differences across groups in peak ELISpot responses or in intracellular cytokine responses were analyzed using the Kruskal–Wallis test with Dunn’s multiple comparison post-test. Similarly, variations between post-prime and post-boost responses measured by intracellular staining were compared for each cytokine, on CD4^+^ and CD8^+^ T cells separately, using Kruskal–Wallis test with Dunn’s correction for multiple comparisons. This non-paired analysis was chosen to gain some statistical power over a matched-pairs test, as several data points were missing from this dataset. Comparisons between study groups of the T cell phenotype distribution of peak CD4^+^ and CD8^+^ T cell responses was achieved by a non-parametric partial permutation test using 10,000 iterations in SPICE (24). All statistical tests were two-tailed and a *p*-value of less than 0.05 was considered significant.

### Ethical Considerations and Regulatory Study Approval

An independent data safety monitoring board (DSMB) was appointed before the trial began to provide oversight and to review the safety data reports as the trial progressed. An experienced local pediatrician served as the local safety monitor (LSM) and, along with the DSMB, reviewed safety data for 16- and 8-week-old infants before commencing vaccination in the 1-week-old group. The trial was conducted according to ICH Good Clinical Practice guidelines and the Declaration of Helsinki principles; and was monitored by an external organization (Appledown Clinical Research Ltd., UK). The trial protocol was also approved by the Gambia Government/Medical Research Council (MRC) Joint Ethics Committee, The Gambia Medicines Board and Oxford Tropical Research Ethics Committee (OXTREC Number: 7-14). This clinical trial was registered with http://clinicaltrials.gov (NCT02083887) and the Pan African Clinical Trials Registry, www.pactr.org (PACTR 201402000749217).

## Results

### Recruitment

Seventy-two infants and neonates were screened for eligibility and 65 eligible infants were enrolled, randomized, vaccinated according to randomization list and followed up for 252 days. The participant flow chart is shown in Figure 1, while the baseline demographic and laboratory parameters are summarized for each group in Table 1.

**Table 1:** Baseline demographic and laboratory parameters of study infants.

Age group	16-week old		8-week old		1-week old	
		
Randomized group	Vaccine (*n* = 15)	Control (*n* = 5)	Vaccine (*n* = 15)	Control (*n* = 5)	Vaccine (*n* = 15)	Control (*n* = 10)
		
Sex (female)	7 (47.7%)	3 (60.0%)	9 (60.0%)	2 (40.0%)	12 (80.0%)	3 (30.0%)
		
	Median (IQR)		Median (IQR)		Median (IQR)	
Hemoglobin (g/dl)	10.9 (10.1–12)	10.4 (10.3–10.7)	11.6 (10.9–13)	11.5 (10.8–11.8)	19.1 (16.8–20.4)	18.45 (16.5–20.9)
White cell count (×10^9^/l)	8.4 (7.8–9.3)	10.9 (8.7–14.3)	10.4 (8.7–13.6)	7.4 (6.8–11.2)	18 (13–19.8)	14.95 (13.9–17)
Neutrophil count (×10^9^/l)	1.9 (1.2–2.7)	1.4 (0.8–2.3)	3.4 (2.9–5.2)	1.6 (1.4–3.6)	10.8 (6.8–13.3)	5.1 (4.2–9.4)
Lymphocyte count (×10^9^/l)	5.5 (4.4–6.5)	7.7 (6.0–10.4)	5.7 (4.2–8.2)	4.9 (4.4–8.4)	5.4 (3.8–5.3)	7.0 (5.0–10.2)
Platelet count (×10^9^/l)	403 (282–559)	591 (552–667)	295 (250–389)	271 (255–272)	185 (158–233)	136.5 (108–239)
Alanine transaminase (ALT) (μmol/l)	26 (17–32)	24 (24–26)	18 (15–25)	21 (20–33)	21 (16–29)	19.5 (14–25)
Creatinine (U/l)	11 (10–15)	13 (13–14)	21 (18–23)	21 (19–21)	55 (51–57)	53.5 (43–64)

### Safety and Reactogenicity

Following ChAd63 ME-TRAP coadministration with EPI vaccines in the 16-week-old cohort, fever was documented in similar numbers of infants (11/15, 73%) compared to the control (EPI vaccines only) group (80%, 4/5, *p* = 0.63 by FET) (Table 2). Excessive crying was recorded in 60% (3/5) and 40% (8/15) of the controls and vaccinees, respectively (*p* = 0.60 by FET). Episodes of discoloration and swelling at EPI site were similar in frequency (2/15 vs. 1/5) (Table 3).

**Table 2:** Systemic solicited adverse events during 3-day follow-up after each study vaccination.

	Post-ChAd63 multiple epitope string thrombospondin-related adhesion protein (ME-TRAP)						Post-MVA ME-TRAP					
Age group	16 weeks		8 weeks		1 week		16 weeks		8 weeks		1 weeks	
*n* (%)	Vaccine	Control	Vaccine	Control	Vaccine	Control	Vaccine	Control	Vaccine	Control	Vaccine	Control
Reported fever	1 (6.7)	5 (100.0)	7 (46.7)	5 (100.0)	14 (93.3)	7 (70.0)	2 (13.3)	0	4 (26.7)	2 (40.0)	15 (100.0)	7 (70.0)
Documented fever	11 (73.3)	4 (80.0)	10 (66.7)	1 (20.0)	8 (53.3)	1 (10.0)	10 (66.7)	1 (20.0)	12 (80.0)	2 (40.0)	9 (60.0)	1 (10.0)
Excessive crying	8 (53.3)	3 (60.0)	6 (40.0)	4 (80.0)	10 (66.7)	4 (40.0)	2 (13.3)	0	10 (66.7)	2 (40.0)	9 (60.0)	3 (30.0)
Refusal of feed	1 (6.7)	1 (20.0)	1 (6.7)	0	1 (6.7)	0	0	0	3 (20.0)	0	1 (6.7)	0
Vomiting	1 (6.7)	0	2 (13.3)	2 (40.0)	2 (13.3)	1 (10.0)	0	0	6 (40.0)	0	1 (6.7)	2 (20.0)
Diarrhea	8 (53.3)	1 (20.0)	3 (20.0)	1 (20.0)	5 (33.3)	2 (20.0)	0	0	5 (33.3)	0	3 (20.0)	1 (10.0)

*This table shows that following ChAd63 and MVA ME-TRAP administrations, fever, excessive crying, refusal of feed, vomiting, and diarrhea were more frequently observed among 1- and 8-week-old cohorts than 16-week-old cohort*.

**Table 3:** Local solicited adverse events during 3-day follow-up after each study vaccination.

	Post-ChAd63 multiple epitope string thrombospondin-related adhesion protein (ME-TRAP)						Post-MVA ME-TRAP					
Age group	16 weeks		8 weeks		1 week		16 weeks		8 weeks		1 week	
*n* (%)^a^	Vaccine	Control	Vaccine	Control	Vaccine	Control	Vaccine	Control	Vaccine	Control	Vaccine	Control
EPI site pain	1 (6.7)	0	10 (66.7)	2 (40.0)	3 (20.0)	4 (40.0)	0	0	2 (13.3)	0	4 (26.7)	5 (50.0)
Limitation of movement at EPI site	0	0	0	0	0	0	0	0	0	0	1 (6.7)	0
Study vaccine site pain	0	0	0	0	0	0	1 (6.7)	0	0	0	0	0
EPI site discoloration	2 (13.3)	1 (20.0)	4 (26.7)	0	1 (6.7)	2 (20.0)	0	0	0	0	2 (13.3)	2 (20.0)
Study vaccine site discoloration	0	0	0	0	0	0	1 (6.7)	0	1 (6.7)	0	0	0
EPI site swelling	1 (6.7)	1	7 (46.7)	1 (20.0)	0	2 (20.0)	0	0	6 (40.0)	2 (40.0)	1 (6.7)	2 (20.0)
Study vaccine site swelling	0	0	0	0	0	0	1 (6.7)	0	0	0	0	0

*^a^For 16- and 8-week groups, n = 15 for vaccine arm and n = 5 for control arm, while for 1-week group, n = 15 for vaccine arm and n = 10 for control arm*.

*This table shows that pain, discoloration, and swelling at EPI injection sites were more frequently reported in participants who received EPI with study vaccines across all age groups*.

A fatal SAE was recorded in a female study infant 5 days after coadministration of ChAd63 ME-TRAP with EPI vaccines. Vital signs immediately post-vaccination and during the 3-day post-vaccination follow-up were within acceptable ranges. Mother attended a social event with the child 5 days after receiving the vaccines and the child reportedly died while sleeping. Verbal autopsy report suggested that the probable cause of death was sudden infant death syndrome, and this was considered not likely to be related to the study vaccine.

After vaccination with MVA ME-TRAP, as expected, a higher proportion of vaccinees had systemic AEs than the controls (fever: 10/15, 67% vs. 1/5, 20%, *p* = 0.09; vomiting: 2/15, 13% vs. 0/5, 0%, *p* = 0.53 using FET) (Table 2). For both ChAd63 and MVA ME-TRAP, the onset of most of the AEs was 24 h after vaccination and resolution was within 48 h. The AEs were of mild intensity and were considered possibly related to the study vaccines.

In the 8-week-old group, all AEs reported after vaccination with EPI vaccines alone or with ChAd63 ME-TRAP were mild and resolved within 1 day of onset, with fever being the most frequently reported among those in the vaccine arm (10/15, 67%) (Table 2). Similarly, following MVA ME-TRAP vaccination, fever was the most commonly reported AE in the vaccine arm (12/15, 80% vs. 2/5, 40%, *p* = 0.13 using FET, Table 2). Similarly, excessive crying, refusal of feeds, and vomiting were more frequently observed in the control group post-ChAd63 administration, while these symptoms were reported more frequently in the vaccine recipients post-MVA vaccination. These were of mild intensity (Grade 1) and were considered to be possibly related to the study vaccines.

More systemic AEs were observed in the neonates who received ChAd63 ME-TRAP with EPI vaccines than those who received EPI vaccines only (fever: 8/15, 53% vs. 1/10, 10%, *p* = 0.03 using FET, Table 2). Similarly, following MVA ME-TRAP vaccination, frequently observed AEs in the vaccine arm included fever (60 vs. 10%, *p* = 0.02) and excessive crying (60 vs. 30%, *p* = 0.12) (Table 2). The AEs were mild in intensity and were observed 24 h after vaccination and resolved within 48 h. Also, higher proportion of study infants in the control arm had pain at EPI injection site (50 vs. 26.7%, *p* = 0.22), discoloration at EPI injection site (20 vs. 13.3%, *p* = 0.53) and swelling at EPI site (20 vs. 6.7%, *p* = 0.37, using FET) (Table 3).

Prominent among unsolicited AEs during 30 days after each study vaccination were upper respiratory tract infection in 20% of study vaccine recipients among 16-week-old cohort and skin sepsis in 1- and 8-week old cohort (13.3 and 73.3%, respectively) (Table 4). Owing to mild intensity of these symptoms, the participants were clinically investigated to establish the definitive diagnoses and exclude possible complications. The participants with skin sepsis (impetigo) were subsequently treated with full course of antimicrobials on out-patient basis and they made remarkable clinical recovery. Low hemoglobin observed in one neonate (6.7%) in the vaccine group was investigated and found to be due to physiological decline of hemoglobin in early weeks of life. This was corrected with hematinics and the participant made sustained clinical improvement. These events were considered not related to the study vaccines, given that they occurred more than 2 weeks post-vaccinations.

**Table 4:** Incidence of unsolicited adverse events during 30 days after study immunizations.

	Post-ChAd63 multiple epitope string thrombospondin-related adhesion protein (ME-TRAP)						Post-modified vaccinia virus Ankara (MVA) ME-TRAP					
Age group	16 weeks		8 weeks		1 week		16 weeks		8 weeks		1 week	
*n* (%)	Vaccine	Control	Vaccine	Control	Vaccine	Control	Vaccine	Control	Vaccine	Control	Vaccine	Control
Upper respiratory tract infection	3 (20.0)	1 (20.0)	5 (33.3)	0	1 (6.7)	0	0	0	1 (6.7)	0	1 (6.7)	1 (20.0)
Low hemoglobin	0	1 (20.0)	0	0	0	0	0	0	0	0	1 (6.7)	0
Acute conjunctivitis	0	0	0	0	1 (6.7)	0	0	0	0	0	0	0
Pustular scalp sepsis	0	0	0	0	1 (6.7)	1 (20.0)	0	0	0	0	0	0
Skin sepsis	0	0	0	0	2 (13.3)	0	1 (6.7)	0	11 (73.3)	1 (20.0)	0	0
Restlessness	0	0	0	0	1 (6.7)	0	0	0	0	0	0	0
Eye discharge	0	0	0	0	0	0	1 (6.7)	0	0	0	0	0
Moderate bronchiolitis	0	0	0	0	0	0	1 (6.7)	0	0	0	0	0
Tinea capitis	0	0	0	0	0	0	0	0	0	0	1 (6.7)	0

*Skin sepsis (impetigo) was frequently reported in 8-week-old cohort (73.3%) following MVA-ME-TRAP and one neonate post-ChAd63 vaccination. Low hemoglobin observed in another neonate was due to physiological decline in early life*.

A SAE occurred due to hospitalization in a female infant who developed severe bronchopneumonia 4 days after vaccination with MVA ME-TRAP and EPI vaccines. She was managed with intravenous antibiotics and intranasal oxygen. After clinical improvement, she was discharged home 5 days after admission. This event was considered unlikely to be related to the study vaccine.

Except for low hemoglobin reported in two participants (one in vaccine group in 1-week-old cohort and another in control group of 16-week-old cohort), all hematological and biochemical parameters were within normal ranges throughout the follow-up period across the study arms. No participants developed clinical malaria during the study.

### Immunogenicity

#### T Cell Responses to Vaccination

**Figure 2:**
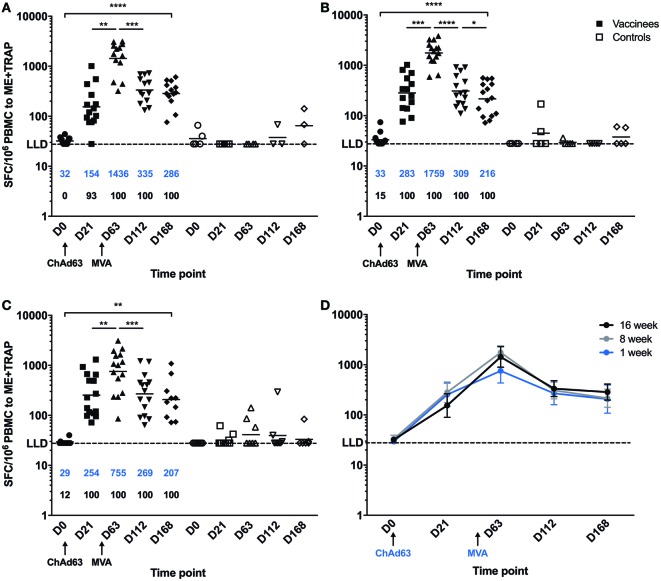
*Ex vivo* IFNγ enzyme-linked immunospot responses to multiple epitope string thrombospondin-related adhesion protein (ME-TRAP) pre- and post-vaccination with ChAd63 and MVA ME-TRAP in Gambian infants. **(A–C)** Scatter plots show individual responses to ME-TRAP in both vaccinees (in black) and controls (in white) of each age group. **(A)** 16-week-old; **(B)** 8-week-old; **(C)** 1-week-old infants. **(D)** Geometric mean ME-TRAP responses in each age group over period of follow-up. LLD, lower limit of detection of the assay. Increases in responses over the time course were analyzed using two-tailed Friedman test. Responses between individual time points were compared within each age group using two-tailed Wilcoxon analysis, *p < 0.05, **p < 0.01, ***p < 0.001, ****p < 0.0001. Lines and numbers in blue denote geometric mean. Numbers shown in black represent the percentage of responders among the vaccinated infants for each time point.

**Figure 3:**
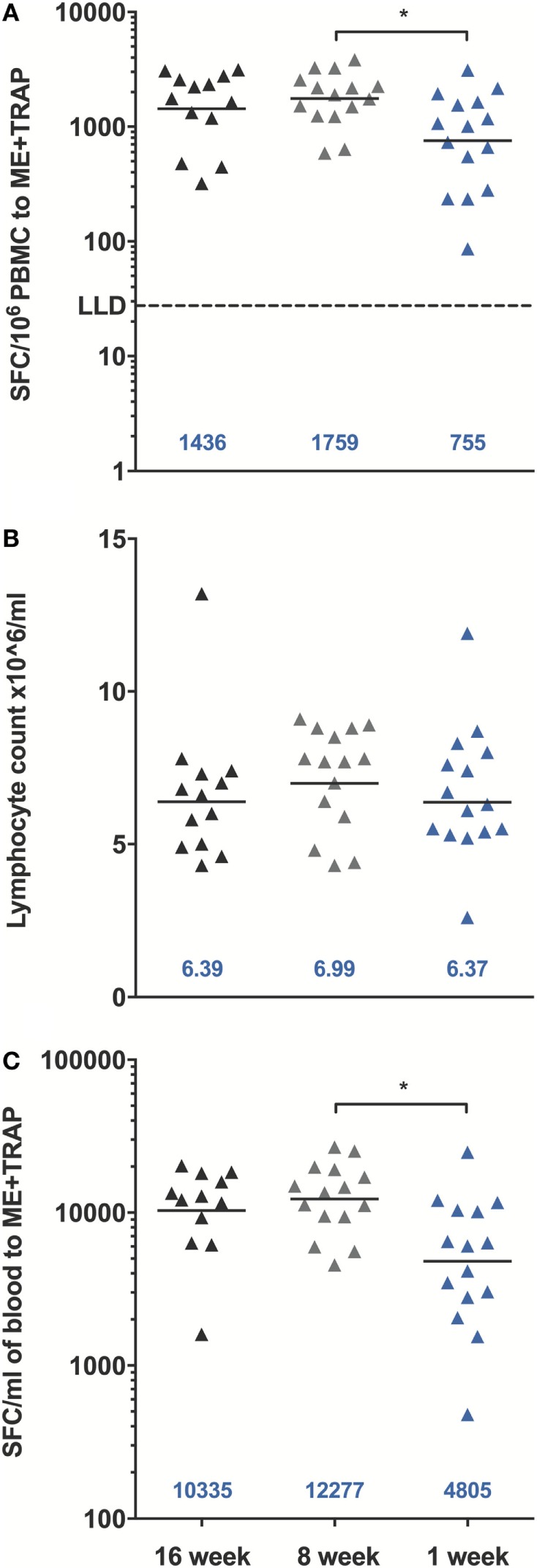
Peak enzyme-linked immunospot (ELISpot) responses to multiple epitope string thrombospondin-related adhesion protein (ME-TRAP) at D63, 7 days post-MVA. **(A)** Peak responses from vaccinated infants expressed per million PBMC. **(B)** Lymphocyte counts per milliliter of blood at D63. **(C)** ELISpot responses expressed per ml of blood. LLD, lower limit of detection of the assay. Peak responses and cell counts were compared across groups using Kruskal–Wallis test with Dunn’s multiple comparison post-test, *p < 0.05. Lines and numbers indicate geometric mean.

A significant increase in ME-TRAP-specific IFNγ responses from baseline was observed 21 days after priming with ChAd63 ME-TRAP in all vaccinees as compared to controls, using an *ex vivo* IFNγ enzyme-linked immunospot (ELISpot) assay (Figures 2A–C). Vaccination with MVA ME-TRAP boosted the magnitude of these responses, which increased significantly from post-priming levels in all age group. One week post-MVA, 100% responder frequency was observed in all age groups and peak geometric mean responses reached 1,436 [with 95% confidence interval (95% CI 1,190–2,388), 1,759 (95% CI 1,467–2,515), and 755 (95% CI 622–1,568) SFC per million PBMC for the 16-, 8-, and 1-week-old infants, respectively, as compared with pre-boost responses of 154 SFC (95% CI 84–395), 283 SFC (95% CI 209–546), and 254 SFC (95% CI 182–590) per million PBMC, *p* = 0.0012, *p* = 0.0002, *p* = 0.0043, respectively, two-tailed Wilcoxon test]. IFNγ T cell responses were maintained significantly above post-priming levels up to 112 days following boost vaccination in all vaccinated groups (day 168, Figure 2D), while responses in controls remained below 100 SFC throughout follow-up. Because lymphocyte counts per milliliter of blood can vary with age (14, 25), we expressed peak responses 1 week post-boost (at day 63) as SFC per milliliter of blood by combining lymphocyte counts collected at this time point during routine hematology tests (Figure 3). Using this unit, highest responses to vaccination with ChAd63 MVA ME-TRAP were still observed in the infants aged 8 weeks at first vaccination (*p* > 0.99 and *p* = 0.015 when compared to the 16- and 1-week-old infants, respectively, two-tailed Kruskal–Wallis test with Dunn’s correction for multiple tests) (Figure 3C), as lymphocyte counts did not vary significantly between groups (Figure 3B).

**Figure 4:**
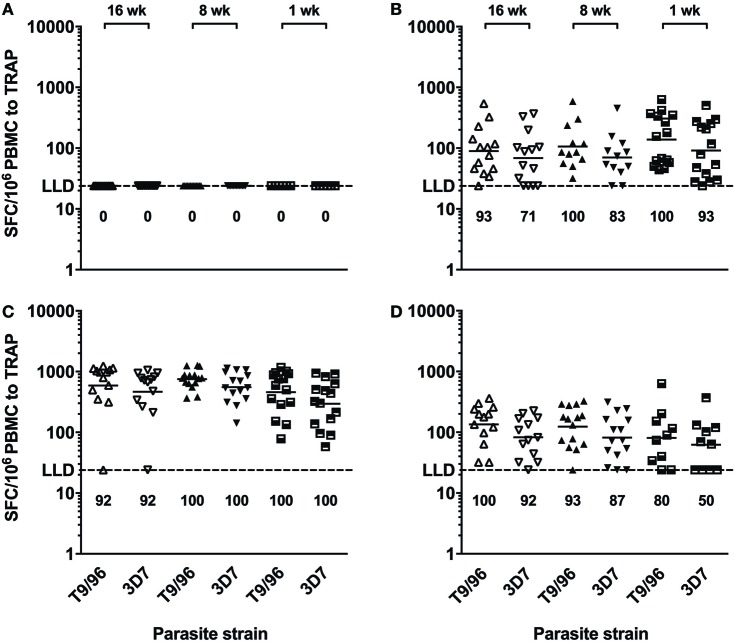
T cells induced by vaccination with thrombospondin-related adhesion protein (TRAP) antigen from *P. falciparum* T9/96 strain are highly cross-reactive. Scatter plots show individual *ex vivo* IFNγ enzyme-linked immunospot responses to TRAP peptides from T9/96 and 3D7 strains, respectively. **(A)** Responses pre-vaccination. **(B)** Responses 3 weeks post-prime with ChAd63 multiple epitope string thrombospondin-related adhesion protein (ME-TRAP). **(C,D)** Responses at day 63 and day 168, 7 and 112 days post-boost with MVA ME-TRAP, respectively. Numbers indicate the percentage of positive responses for each age group at each time point for both parasite strains. LLD, lower limit of detection of the assay. Lines represent geometric mean.

IFNγ ELISpot responses to the TRAP antigen were always significantly higher than those to the ME string (Figure S2 in Supplementary Material) and were relatively broad (Figure S3 in Supplementary Material). Responses to TRAP from heterologous 3D7 *P. falciparum* strain were of comparable magnitude as compared to the corresponding responses to TRAP from homologous T9/96 vaccine strain throughout follow-up in all age groups (Figure 4). In addition, peak responses, 1 week post-MVA, to both strains strongly correlated (*r* = 0.813, *p* = 0.0012; *r* = 0.770, *p* = 0.0012; *r* = 0.986, *p* < 0.0001, in the 16-, 8-, and 1-week-old groups, respectively, Spearman correlation test), showing induction of highly cross-reactive T cells by vaccination with the T9/96 strain.

#### TRAP-Specific T Cell Response Cytokine Profile

**Figure 5:**
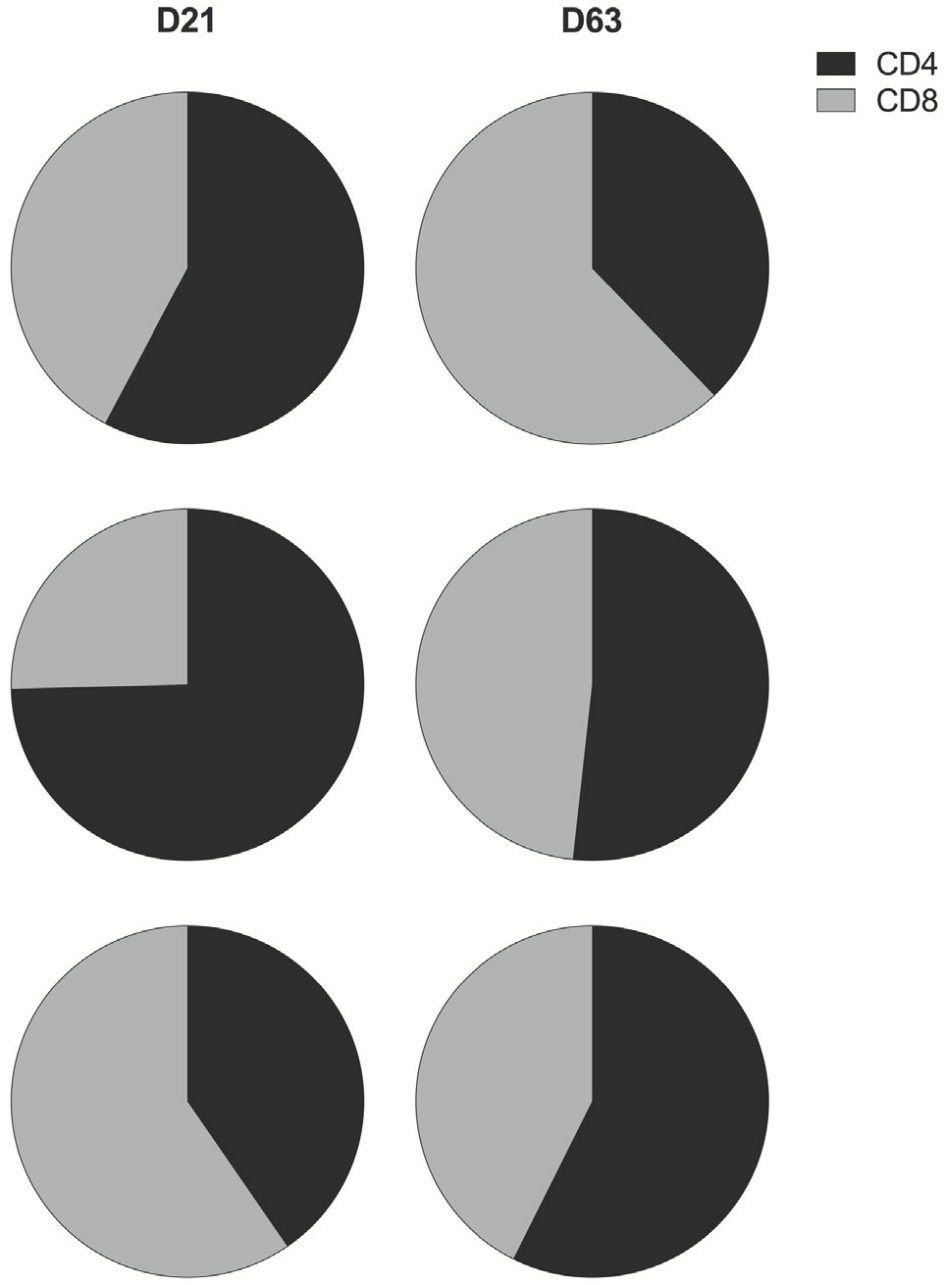
CD4^+^ and CD8^+^ T cell contribution to total IFNγ response to thrombospondin-related adhesion protein (TRAP). Pie charts display the proportion of IFNγ produced by CD4^+^ (black) and CD8^+^ (gray) T cells as a fraction of the total IFNγ response to TRAP post-prime and post-boost vaccination. Here, any negative response was excluded from analysis.

In all groups, prime-boost immunization with ChAd63 MVA ME-TRAP induced both CD4^+^ and CD8^+^ TRAP-specific T cells (Figures S4 and S5 in Supplementary Material). IFNγ production by both T cell subsets was detected in all age groups 3 weeks post-prime vaccination. Boost immunization with MVA induced a marked increase in the magnitude of these responses, although it only reached significance for the CD4^+^ T cell response in the oldest and the youngest infants (*p* = 0.002 and *p* = 0.01, respectively, Kruskal–Wallis test, Figures S4B–D in Supplementary Material) and for the CD8^+^ T cell response of the 16- and 8-week olds (*p* = 0.001 and *p* = 0.027, respectively, Kruskal–Wallis test, Figures S5B–D in Supplementary Material). Prior to boosting, IFNγ was largely secreted by CD4^+^ T cells, except in the youngest age group where CD8^+^ T cells were the main IFNγ producers. By contrast, after boosting with MVA, the contribution to total IFNγ response to TRAP was more evenly distributed between both T cell populations in all age groups (Figure 5).

**Figure 6:**
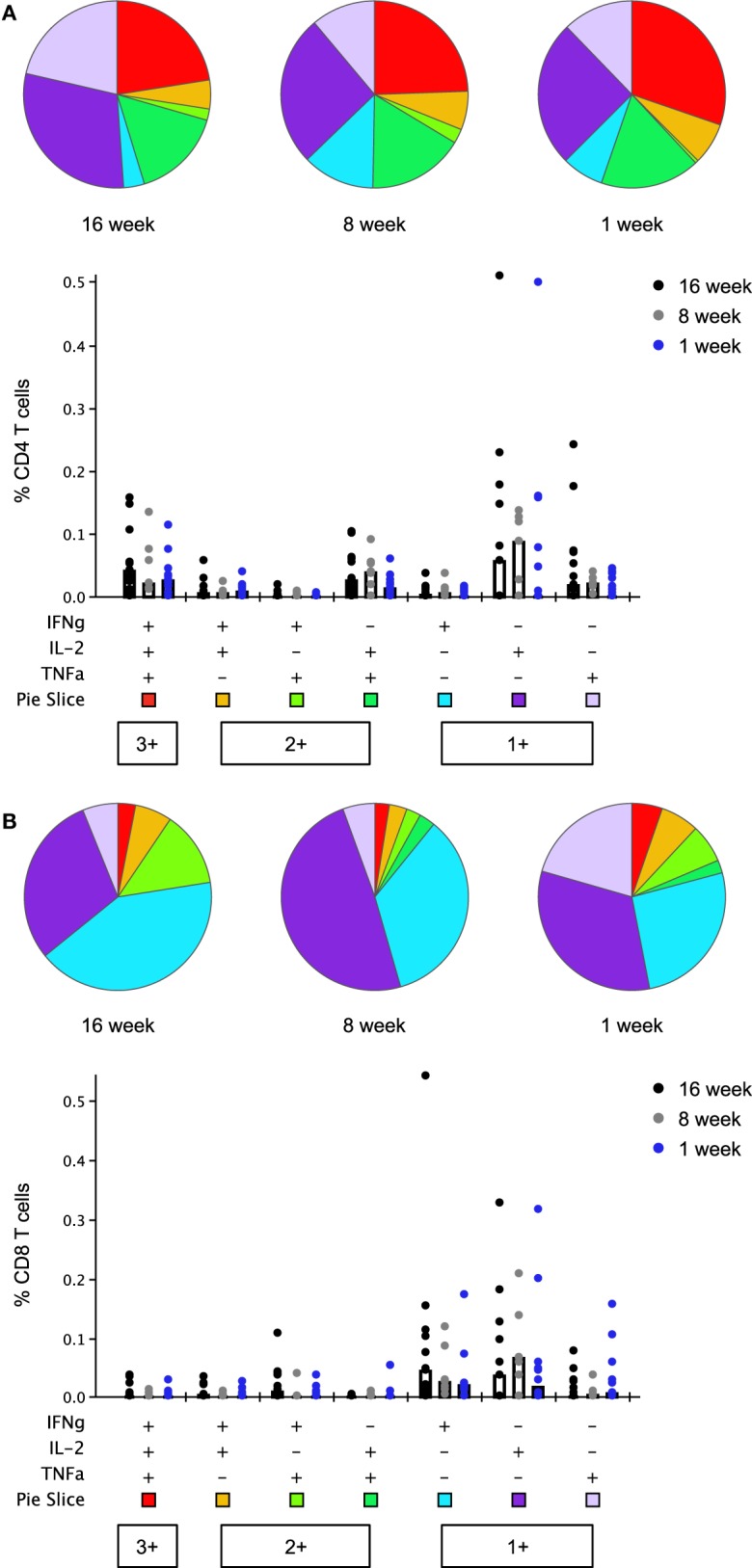
Multifunctionality of peak CD4^+^ and CD8^+^ T cell responses to thrombospondin-related adhesion protein (TRAP) at day 63 (1 week post-boosting). Cytokine production from CD4^+^ **(A)** and CD8^+^ **(B)** T cells was analyzed using Boolean gating analysis. Pie charts illustrate the relative frequency of each combination of cytokines, indicated in the bar chart below, as a fraction of the total cytokine response. Bar charts show the absolute frequency of each subset in response to stimulation with TRAP peptides, compared between each age group. Bars show median responses in this case as geometric mean is not available in SPICE. Any response which was less than or equal to the background (unstimulated) response was replaced by lower limit of detection value in bar chart and excluded from pie charts.

Thrombospondin-related adhesion protein-specific T cells comprised a mixture of T cell subsets with distinct effector functions in both the CD4^+^ and CD8^+^ T cell populations (Figure 6). Seven days after boosting with MVA, the functional profile of the peak CD4^+^ and CD8^+^ T cell responses was similar between study groups [no significant difference in the phenotype distribution using the non-parametric partial permutation test in Spice (NPPPTS)] (24). The majority of cytokine-secreting T cells produced a single cytokine, T cell subsets expressing two or three cytokines simultaneously were also observed in all age groups (Figure S6 in Supplementary Material). As previously observed, CD4^+^ T cells displayed a more polyfunctional phenotype than CD8^+^ T cells, across all three age groups (*p* = 0.05, *p* = 0.02, *p* = 0.05 in the 16-, 8-, and 1-week-old groups, respectively, NPPPTS).

A population of monofunctional CD8^+^ T cells, positive for IFNγ, but not for IL-2 or TNFα, previously found associated with delay to patency following controlled human malaria infection of malaria-naïve adult volunteers using this vaccine (9) was detected in all three infant groups (Figure S7 in Supplementary Material). Boosting with MVA expanded this response, although this increase in magnitude only reached significance in the 16- and 8-week-old infants (*p* = 0.002 and *p* = 0.016, two-tailed Kruskal–Wallis test); peak responses at day 63 did not differ significantly between groups. This was accompanied by a noticeable rise in the percentage of responders, irrespective of the age group. Positive responses were observed in 92.3, 85.7, and 66.7% of infants at day 63 as compared to 33.3, 33.3, and 53.3% at day 21 in the 16-, 8-, and 1-week-old groups, respectively.

#### Anti-TRAP IgG Responses to Vaccination

**Figure 7:**
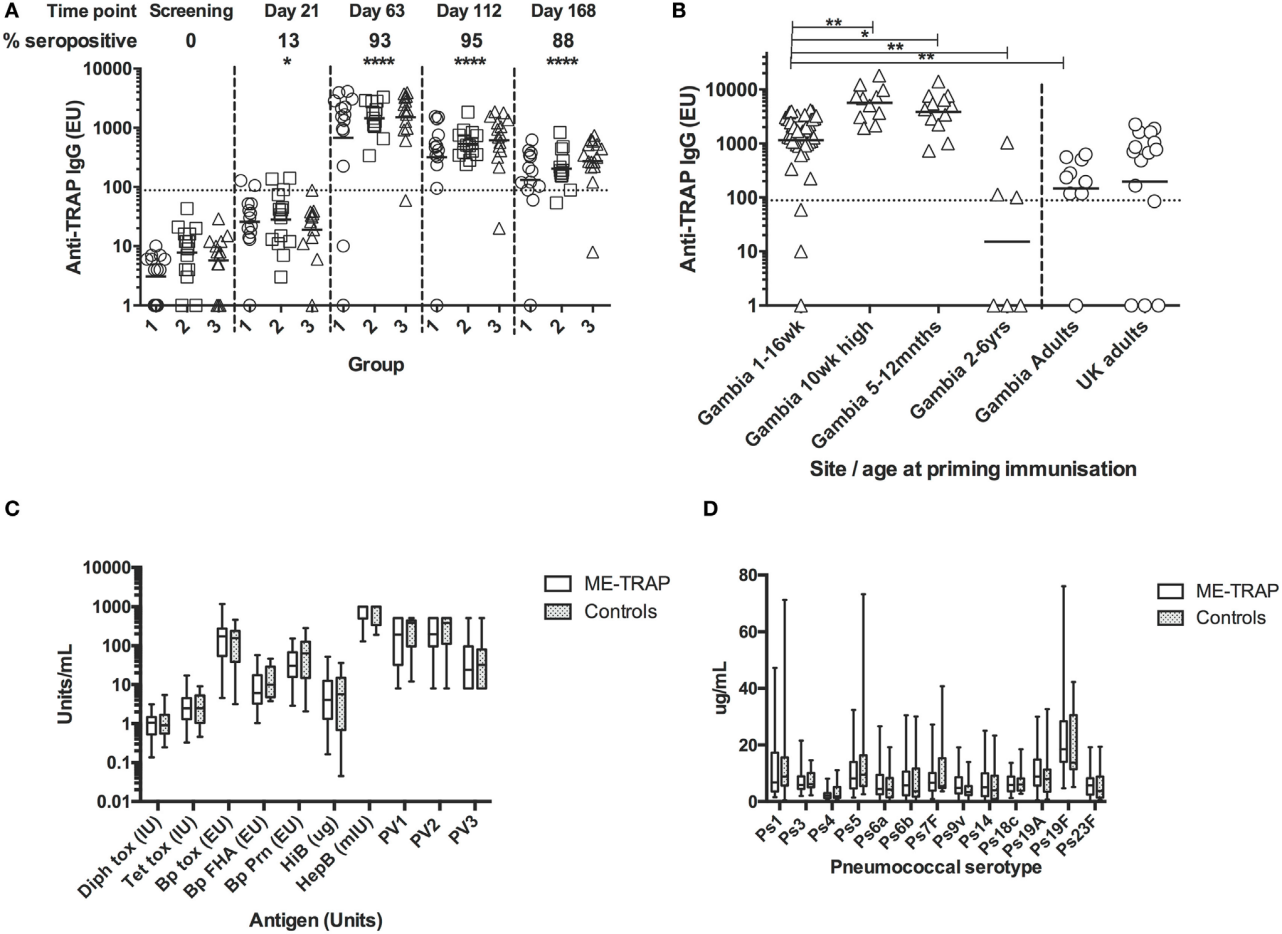
Antibody responses to Expanded Program on Immunization (EPI) and multiple epitope string thrombospondin-related adhesion protein (ME-TRAP) vaccination. **(A)** IgG responses to vaccination with ChAd63 ME-TRAP (day 0) and MVA ME-TRAP (day 56). Scatter plots show peak responses post-prime (day 21) and post-boost (day 63). Group 1—16 weeks, group 2—8 weeks, and group 3—1 week old at first vaccination. All comparisons use Kruskal–Wallis with Dunn’s post-test, comparing responses at day 0 with post-vaccination responses for groups 1–3 combined. No significant differences between groups were observed at any time point. **(B)** Comparison of peak IgG responses with data from previous trials (14). Responses in all groups combined from the present study were compared to those from a study in the same population that received the same dose and vaccine regimen in the absence of EPI coadministration. **(C,D)** Responses to EPI vaccination at 24 weeks, 8 weeks after last primary EPI, among ME-TRAP vaccinees and controls for diphtheria, tetanus, pertussis, *Haemophilus influenza B*, Hepatitis B, and polio serotypes 1–3 **(C)** and pneumococcal immunization (PCV 13) by serotype **(D)**. No significant differences between groups were observed (*p < 0.05, **p < 0.01, ****p < 0.001).

Pre-vaccination IgG titers to TRAP were below the detection limit in all participants (Figure 7A). After vaccination with ChAd63 ME-TRAP, titers increased significantly (*p* < 0.05, Kruskal–Wallis test) with 13% of participants becoming seropositive by day 21. There were no significant differences in antibody response between age groups at any time. Boosting with MVA ME-TRAP increased the seropositivity rate across all groups to 93% at day 63 and titers remained significantly above pre-vaccination levels at 8 and 16 weeks after boosting (*p* < 0.0001, Kruskal–Wallis test). 88% of participants remained seropositive at the last time point measured.

Titers in this trial were compared to titers measured in previous trials of this vaccine in adults and older infants, detected using the same ELISA assay, reagents and operator (10, 13). All groups received 5 × 10^10^ vp ChAd63, with adults receiving 2 × 10^8^ pfu of MVA and all other groups receiving 1 × 10^8^ pfu of MVA. One week after boosting, titers in all groups were strikingly and significantly higher than those in Gambian adults and children aged 2–6 years (Figure 7B, *p* < 0.01, Kruskal–Wallis test). Responses were significantly lower in all three groups than in previous studies in infants aged 5–12 months (*p* < 0.05) or 10 weeks of age without EPI vaccine coadministration (*p* < 0.01, Kruskal–Wallis test), identifying these age groups as preferred ages for use of vectored vaccines for antibody induction. The substantially higher antibody immunogenicity in several young age groups in Africa compared to UK adults (Figure 7B) cautions that some phase Ia trials in adults may importantly underestimate the potency of vectored vaccines in infant target populations.

#### IgG Responses to EPI Vaccines

Responses to EPI vaccines, 8 weeks post-primary series, were not significantly different between vaccinees and controls although the study was not powered to detect modest significant differences (Figures 7C,D).

## Discussion

This clinical trial evaluated the responses to ChAd63 MVA ME-TRAP when given simultaneously with EPI vaccines among healthy Gambian infants and neonates. Vaccines were co-administered as early as 1 week of age, as BCG and the first doses of hepatitis B and OPVs are administered at this time point.

Adverse events observed in the coadministration groups were similar to those reported in our previous studies of these vectored vaccines, where fever was the most frequently observed in the 10-week-old group and all vaccine-related AEs were mild and resolved within 1 day after onset of symptoms (13). Not surprisingly, a significantly higher proportion of infants who received ChAd63 MVA ME-TRAP with EPI vaccines had fever compared to those who received EPI vaccines alone. This finding may be explained by the concurrence of inflammatory processes as a result of multiple injections. Similarly, pain and discoloration at EPI injection sites were more frequently reported in participants who received EPI with study vaccines across all age groups while swelling at EPI sites were seen in participants who received EPI with MVA study vaccine. Nevertheless, these symptoms were of mild intensity and resolved within 48 h post-vaccination.

The increased frequency of fever observed following administration of study vaccines in 1-week-old cohort was notably Grade 1 (of mild intensity) and these resolved within 24 h without use of any medication. There were also no associated danger signs such as refusal of feeds, abnormal cry and convulsion; hence full septic work-up was not done. Given that low grade fever is not unexpected in neonates whose immune system is immature, observing Grade 1 fever over a short duration in this age group lends credence to the safety and tolerability of the study vaccines. However, given the small sample size of the neonates studied, further evaluation is required in larger sample of this cohort. Furthermore, the preponderance of unsolicited symptoms such as skin sepsis observed among study vaccine recipients in 1- and 8-week-old cohorts was clinically investigated and found to be caused by poor hygienic practices by participants’ mothers. The participants fully recovered following improved hygiene and a course of antibiotics. Overall, these observations show that coadministration of ChAd63 MVA ME-TRAP with EPI vaccines is safe and well tolerated in Gambian infants and neonates.

As in previous studies, we observed induction of substantial and durable T cell responses maintained beyond 112 days post-MVA, which comprised both CD4^+^ and CD8^+^ T cells capable of secreting multiple cytokines, with similar phenotypes to those seen in older infants, children, and adults (11, 13, 14). Importantly, these high levels of effector cellular immunity, as measured by ELISpot, with or without adjustment of T cells per million PMBC to T cells per milliliter of blood, are as high or higher than those observed in UK adult vaccinees protected against five bite malaria sporozoite challenge (9) and those observed in Kenyan adult vaccinees showing efficacy against natural infection (12).

Although responses from the 1-week olds were generally lower than those seen in the older infants, probably due to age-related differences in immunity, peak responses 1 week following boost vaccination in this age group were also significantly higher than those previously achieved in Gambian adults (11) as well as in older children in The Gambia and Burkina Faso (14) using the same vaccine approach, further demonstrating that viral vectored vaccines represent an effective mean of triggering cellular immunity in early life. Overall, these data further support previous findings of increase, and not decrease, in cellular immunogenicity in young infants, with the highest responses induced in the 8-week olds being almost identical to those of 10-week olds (12,277 and 12,074 SFC/ml, respectively), who received the same vaccine separately from EPI vaccines (14).

As previously reported, responses to TRAP were broad, indicating the recognition of several potential epitopes within the TRAP antigen (9, 11). Moreover, although TRAP from T9/96 vaccine strain differs by 6.5% AA from 3D7 TRAP sequence (26), peak homologous and heterologous antigen-specific T cell responses were comparable and positively correlated, pointing out a high degree of cross-reactivity. Altogether, these observations confirm that this vaccine should not be substantially affected neither by potentially great genetic variation in HLA types in target populations nor by antigen polymorphism.

Flow cytometry analysis further revealed vaccine-induced cytokine production by both antigen-specific CD4^+^ and CD8^+^ T cells. Importantly, despite major differences (including differences in sensitivity or the use of PBMC vs. whole blood) between the two assays, peak total frequency of IFNγ-producing TRAP-specific CD3^+^ T cells, assessed 1 week post-MVA by intracellular staining, positively correlated with peak IFNγ responses to TRAP measured by ELISpot, suggesting that blood volumes as little as 1.05 ml could be sufficient to measure the vaccine response in young infants.

Priming vaccination elicited predominantly CD4^+^ T cell responses, and while CD8^+^ T cell responses were observed, these required the MVA booster vaccination to reach similar levels. While previous studies have reported age-specific differences in neonatal adaptive immunity with a reduction in infant IFNγ responses to hepatitis B (27) or oral polio (28) vaccines as compared to vaccinated adults, BCG, or DNA vaccinations at birth have been shown to elicit strong T helper type 1 and CD8^+^ T cell responses during neonatal life (29–31). Similarly, our findings illustrate the induction of antigen-specific CD4^+^ and CD8^+^ T cells producing type 1 cytokines, highlighting potent cellular immunogenicity of our vaccine candidate in young infants. This finding confirms that newborns can develop potent cellular immune responses under certain circumstances, as previously observed in infants congenitally infected with *Trypanosoma cruzi* (32) or cytomegalovirus (33), who developed strong CD8^+^ T cell responses.

Multiparameter flow cytometric analysis revealed multiple cytokine-secreting T cell subsets expressing IFNγ, IL-2, and TNFα in all combinations, including a phenotype previously identified as an immune correlate of vaccine-induced protection against heterologous challenge in naïve adults (9). Although these responses were lower in this setting as compared to those seen in UK and Kenyan adults (9, 12), this provides grounds for future studies of vaccine efficacy in pediatric populations.

This viral vector approach also induced substantial IgG antibody responses to TRAP in these infants and neonates, despite a lack of humoral immunogenicity in adults in the same population (11, 14). Peak titers were similarly significantly higher in this cohort than those seen in Gambian children aged 2- to 6-year old, confirming an overall higher immunogenicity in the youngest age groups. The addition of a humoral component of immunity elicited by this vaccine at the pre-erythrocytic stage could be useful, given that viral vectors are predominantly employed for their ability to induce cell-mediated immunity. With our recent observation that vaccine-induced TRAP antibody responses could play a role in reducing liver parasite burden (34), this raises the possibility of an improved vaccine efficacy via synergistic protection in young infants as compared to that observed in adults. The leading malaria vaccine candidate RTS,S shows reduced immunogenicity and associated efficacy in infants aged 6–12 weeks (17), compared with those aged 5–17 months. We observed the same trend in this study, although pre-vaccination titers to TRAP were less frequently observed, suggesting that pre-existing immunity to the vaccine antigen is not the cause in this case.

Given the advantages of integrating a new vaccine within the EPI schedule, including reduced clinic visits, increased immunization coverage, and possible enhanced immunogenicity of the vaccines (15), these benefits must be carefully considered against adverse immunological interference that may be caused by coadministration of T cell inducing vaccines with EPI vaccines. Although our findings support concomitant administration of ChAd63 MVA ME-TRAP with licensed EPI vaccines, these findings need further evaluation in larger non-interference trials.

This study demonstrates that coadministration of ChAd63 MVA ME-TRAP with EPI vaccines is safe and well tolerated and induces potent immune responses against *P. falciparum* antigens. The potency of these vectored vaccines for both T cell and antibody responses in early infancy suggests that vectors may also be useful for targeting other pathogens, such as RSV, where protection in the first months of life is required.

## Ethics Statement

### Ethical Considerations and Regulatory Study Approval

This study was carried out in accordance with the recommendations of the ICH Good Clinical Practice guidelines with written informed consent from all subjects. All parents or carers of subjects gave written informed consent in accordance with the Declaration of Helsinki. The protocol was approved by the Gambia Government/MRC Joint Ethics Committee, The Gambia Medicines Board and Oxford Tropical Research Ethics Committee (OXTREC Number: 7-14). This clinical trial was registered with http://clinicaltrials.gov (NCT02083887) and the Pan African Clinical Trials Registry, www.pactr.org, (PACTR 201402000749217).

An independent DSMB was appointed before the trial began to provide oversight and to review the safety data reports as the trial progressed. An experienced local pediatrician served as the LSM and, along with the DSMB, reviewed safety data for 16- and 8-week-old infants before commencing vaccination in the 1-week-old group. The trial was monitored by an external organization (Appledown Clinical Research Ltd., UK).

## Author Contributions

The authors contributed to the work as listed. Design of research studies: RC, AlN, BF, BC, KB, NV, AL, EC, EI, KE, AH, and MA; conducting experiments: VM, SR, EK, GB, AmN, FO, CB, YJ, SG, and MA; acquiring data: VM, SR, EK, GB, AmN, FO, CB, YJ, SG, and MA; analyzing data: VM, SR, GB, CB, SG, EC, KE, AH, and MA; writing the manuscript: VM, SR, BC, NV, KE, AH, and MA; project management and supervision: RR, FD, OL, BK, BC, KB, NV, AL, EI, AH, and MA.

## Conflict of Interest Statement

The following authors have declared that no conflict of interest exists: VM, SR, EK, AmN, FO, CB, GB, YJ, RR, NV, FD, OL, AL, BF, BK, BC, SG, EC, KE, EI, and MA. AH is a named inventor on patent applications on malaria vectored vaccines and immunization regimens. RC and AlN are employees and/or shareholders in ReiThera, which develops vectored vaccines for malaria and other diseases. The authors declare that the research was conducted in the absence of any commercial or financial relationships that could be construed as a potential conflict of interest. The reviewer AL declared a past collaboration with four of the authors, OL, NV, FD, and AH, to the handling Editor.
