# Supplementary material for: Safety and Immunogenicity of Malaria Vectored Vaccines Given with Routine Expanded Program on Immunization Vaccines in Gambian Infants and Neonates: A Randomized Controlled Trial
Source: Front Immunol. 2017 Nov 20;8:1551. doi: 10.3389/fimmu.2017.01551 (PMC5702785; doi:10.3389/fimmu.2017.01551)
Supplement: Supplementary file 1 [file Data_Sheet_1.PDF]

Supplementary Material

Safety and immunogenicity of malaria vectored vaccines given with routine EPI vaccines in Gambian infants and neonates: a randomized controlled trial

Victorine A. Mensah[1^], Sophie Roetynck[2^], Ebrima K. Kanteh[2], Georgina Bowyer[3], Amy Ndaw[1], Francis Oko[2], Carly M. Bliss[3], Ya Jankey Jagne[2], Riccardo Cortese[4†], Alfredo Nicosia[5,6,7], Rachel Roberts[8], Flavia D'Alessio[9], Odile Leroy[9], Babacar Faye[1], Beate Kampmann[2,11], Badara Cisse[1], Kalifa Bojang[2], Stephen Gerry[10], Nicola K. Viebig[9*], Alison M. Lawrie[8], Ed Clarke[2], Egeruan B. Imoukhuede[8†], Katie J. Ewer[3], Adrian V. S. Hill[3,8], Muhammed O. Afolabi[2]

*** Correspondence:** Nicola K. Viebig: nicola.viebig@euvaccine.eu

1 Supplementary Figures and Tables

1.1 Supplementary Figures

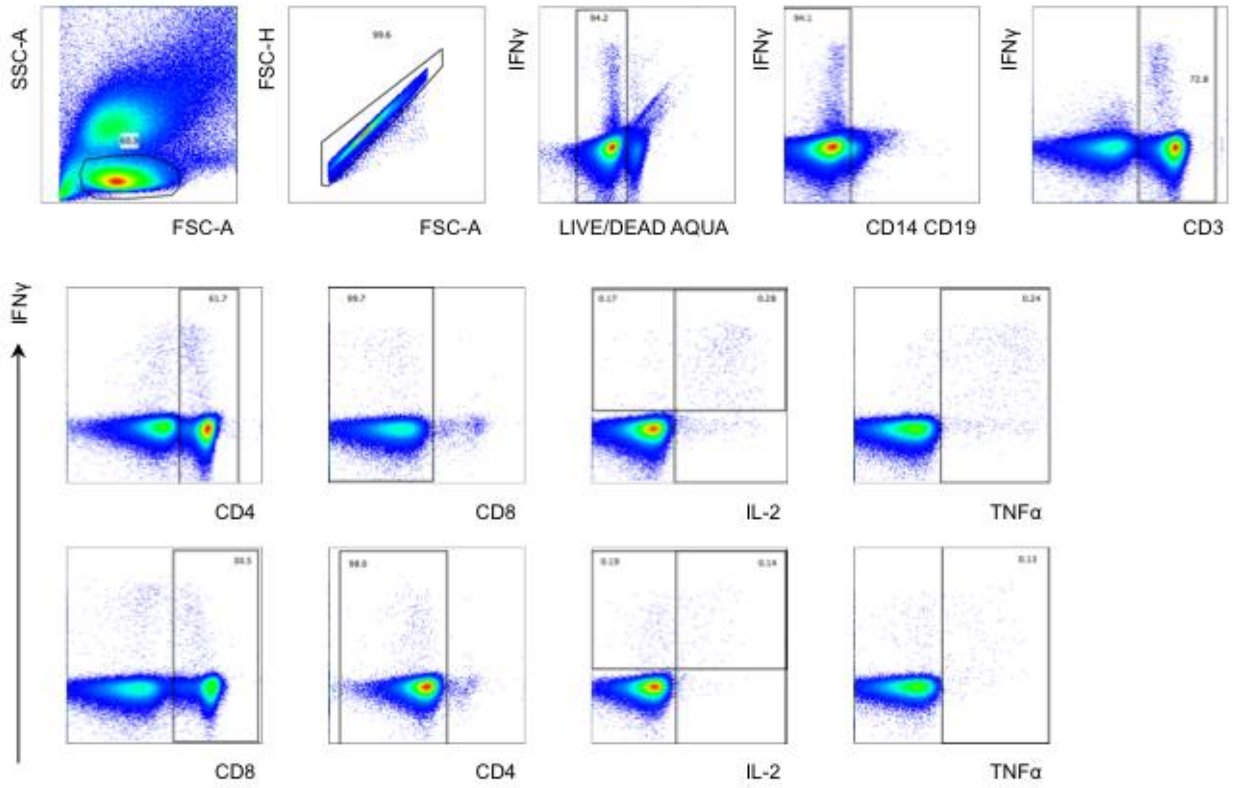

Supplementary Figure S1: Example of T cell responses to TRAP illustrating the hierarchical gating scheme for the analysis of the cytokine response by intracellular staining. Cells were first gated on lymphocytes and singlets. Dead cells, $CD19^+$ B cells and $CD14^+$ monocytes were excluded. Only cells clearly positive for CD3 were included in subsequent analysis. Within the $CD3^+$ population, $CD4^+$ or $CD8^+$ T cells were determined, respectively. The subset expressing each of the analyzed cytokines was determined within both $CD4^+$ and $CD8^+$ gates after exclusion of the $CD4^+$ $CD8^+$ double positives, respectively.

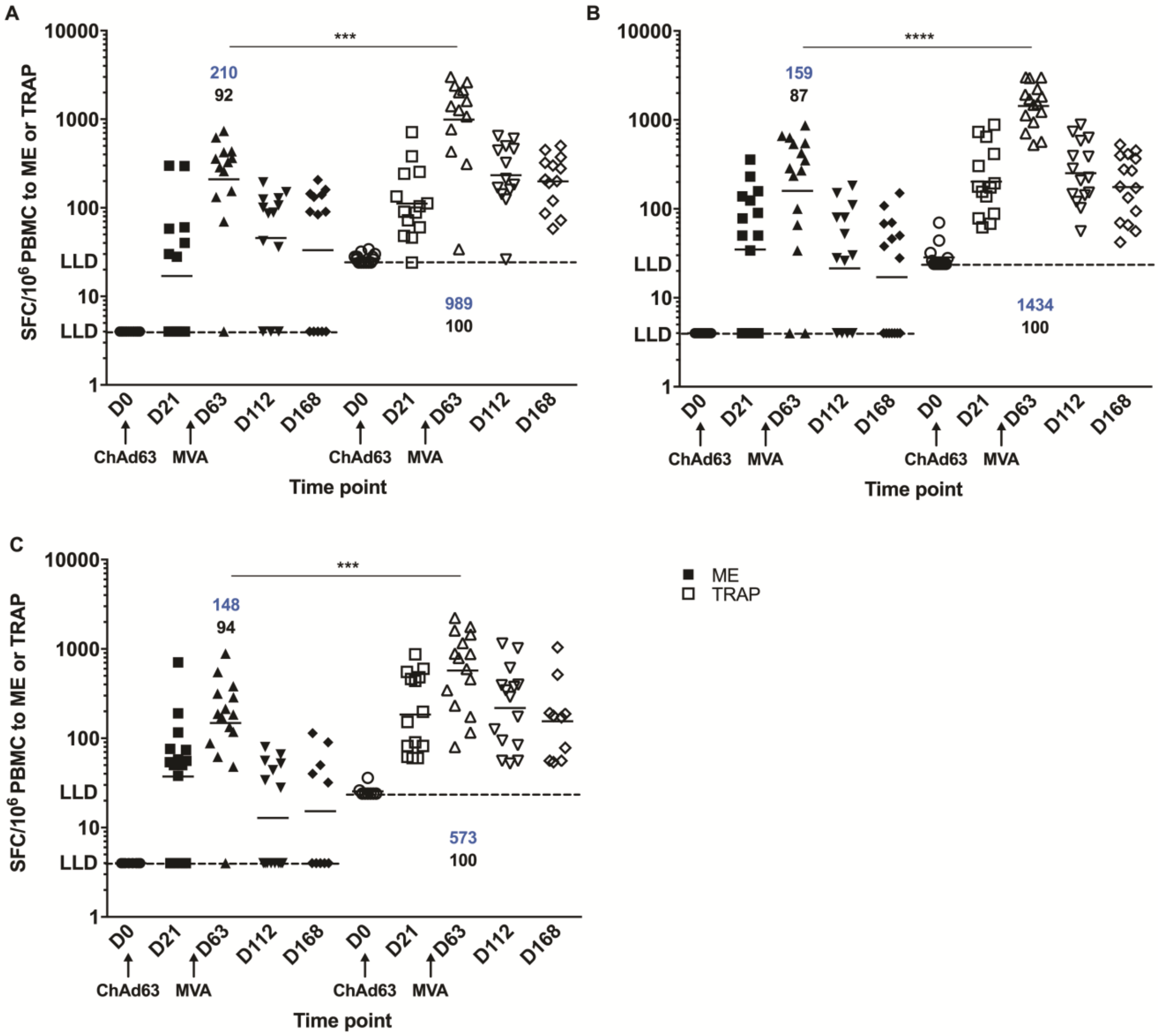

Supplementary Figure S2: *Ex vivo* IFNγ ELISpot responses to the ME string or the TRAP antigen assessed separately pre- and post-vaccination with ChAd63 and MVA ME-TRAP. Scatter plots display vaccinees' individual responses to the ME string (in black) and TRAP peptides (in white) over the course of vaccination and follow-up. A - 16 week old; B - 8 week old; C - 1 week old infants. LLD = lower limit of detection of the assay. Peak responses to ME and TRAP were compared within each age group using 2-tailed Wilcoxon analysis, *p<0.05, **p<0.01, ***p<0.001, ****p<0.0001. Lines and numbers in blue denote geometric mean. Numbers shown in black represent the percentage of responders one week post-MVA.

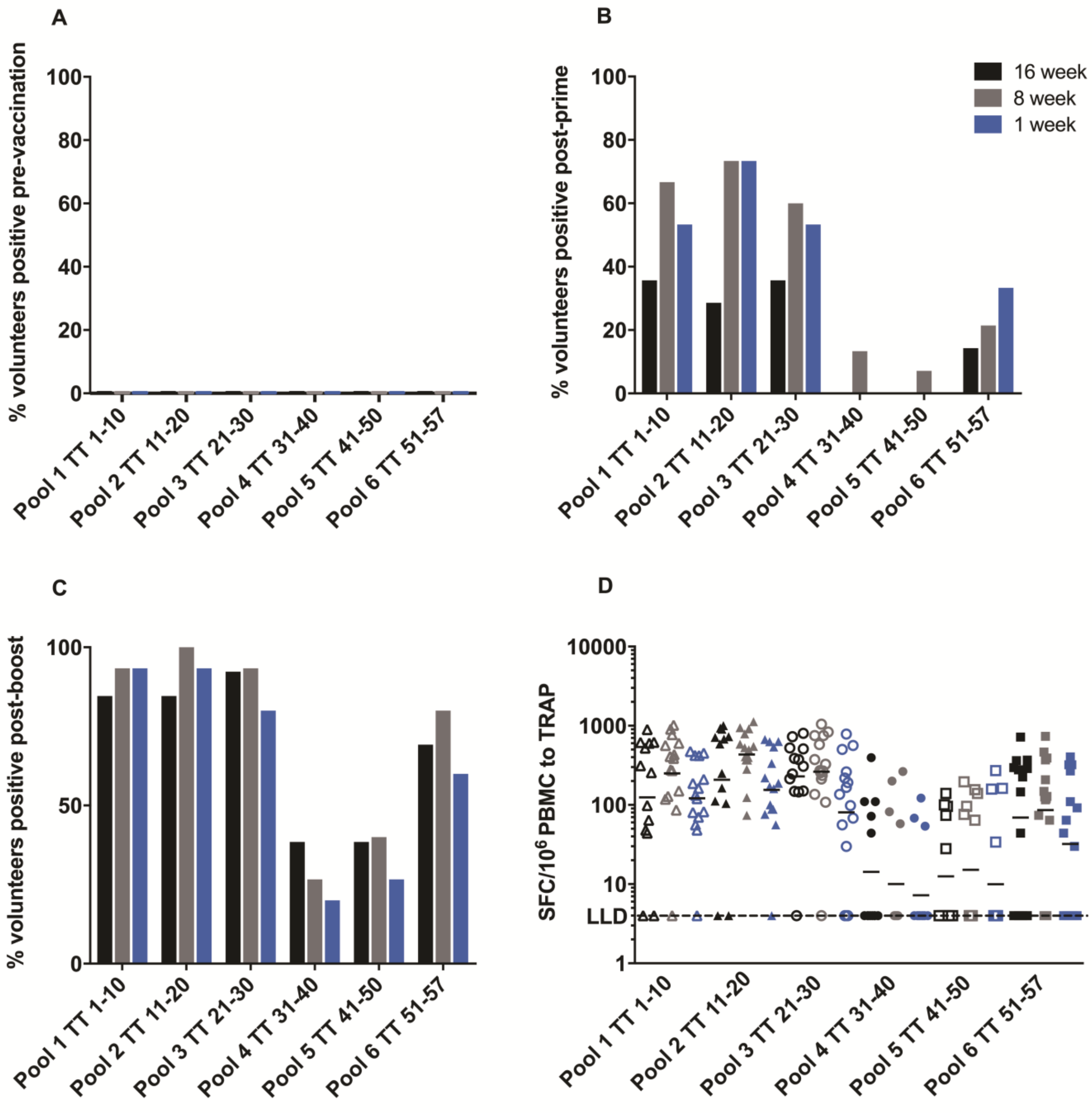

Supplementary Figure S3: Mapping of T cell responses to TRAP antigen following prime-boost vaccination. Using six different pools of overlapping peptides spanning the full length of the TRAP antigen showed that many epitopes were recognized within the TRAP sequence. A-C. Bar charts illustrate the mean percentage of vaccinees responding to each individual peptide pool, A. pre-vaccination, B. 21 days post-priming with ChAd63 ME-TRAP (D21) and C. day 7 post-boosting with MVA ME-TRAP (D63) in each age group. Both the proportion of vaccinees responding to each peptide pool and the breadth of the response rose in all age groups after each vaccination. D Scatter plot shows individual peak IFNγ ELISpot responses to each individual TRAP peptides pool 7 days post-boost with MVA ME-TRAP in each age group. Responses were skewed towards the pools corresponding to the N-terminal (Pool 1-3) and C-terminal (Pool 6) regions of the TRAP antigen,

covering about 80% of the TRAP protein sequence, confirming the recognition of several potential epitopes. LLD = lower limit of detection of the assay. Lines indicate geometric mean.

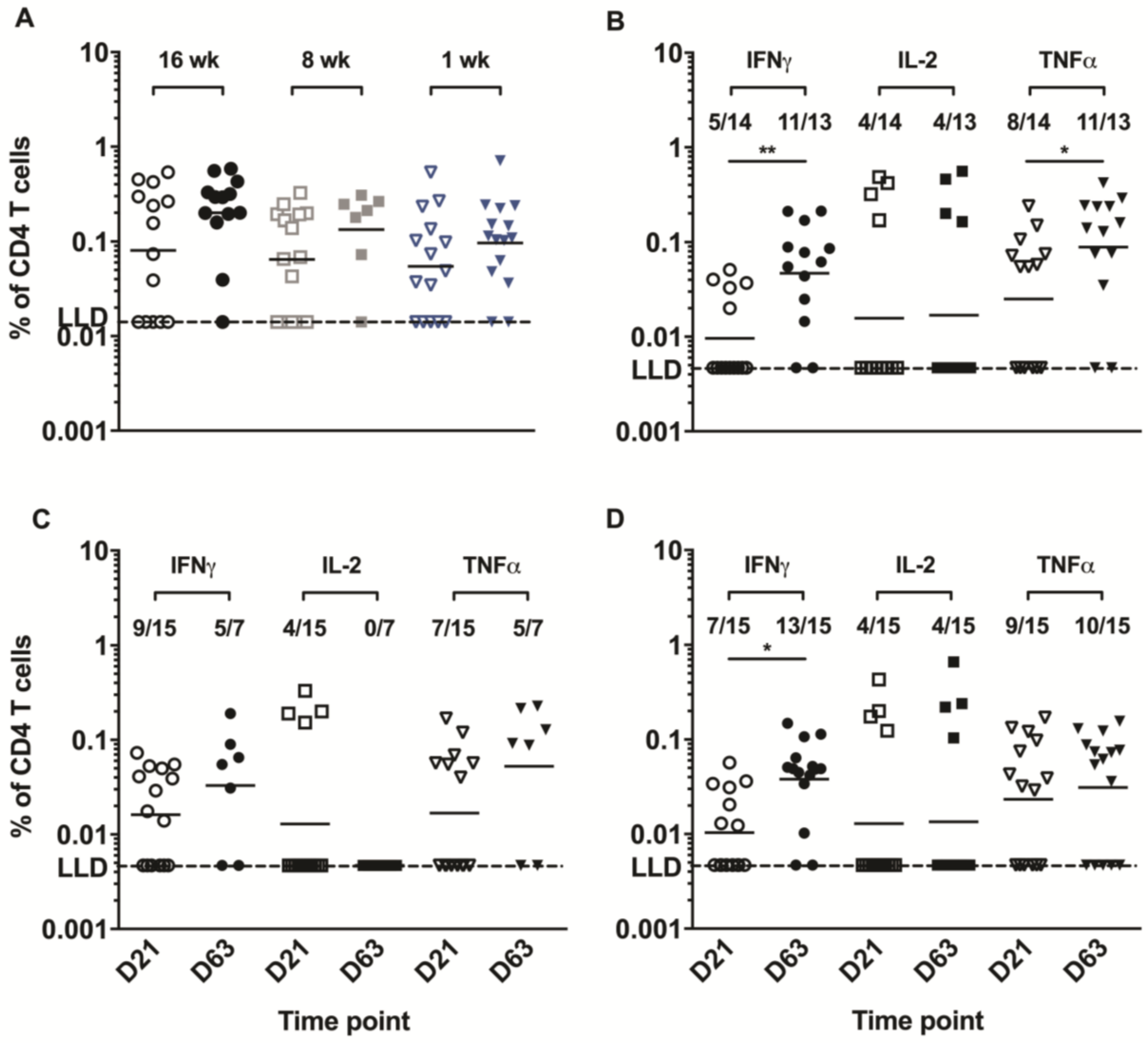

Supplementary Figure S4: TRAP-specific CD4+ T cell response cytokine profile assessed by intracellular staining. Scatter plots show individual responses to TRAP peptides. Cytokine frequencies are shown 21 days post-prime with ChAd63 ME-TRAP (D21) and 7 days post-boost with MVA ME-TRAP (D63) in vaccinees of each age group. A - Total frequency of cytokine positive T cells (i.e. positive for any of the 3 analyzed cytokines); B - responses in the 16 week old; C - responses in the 8 week old; D - responses in the 1 week old group. Bars denote geometric mean. Numbers show the ratio of positive responses to total number of tested samples. LLD = lower limit of detection of the assay. Differences across groups in frequency of cytokine positive CD4+ T cells and individual cytokine responses post-priming and post-boosting within each age group were analyzed using the Kruskal-Wallis test with Dunn's multiple comparison post-test, *p<0.05, **p<0.01.

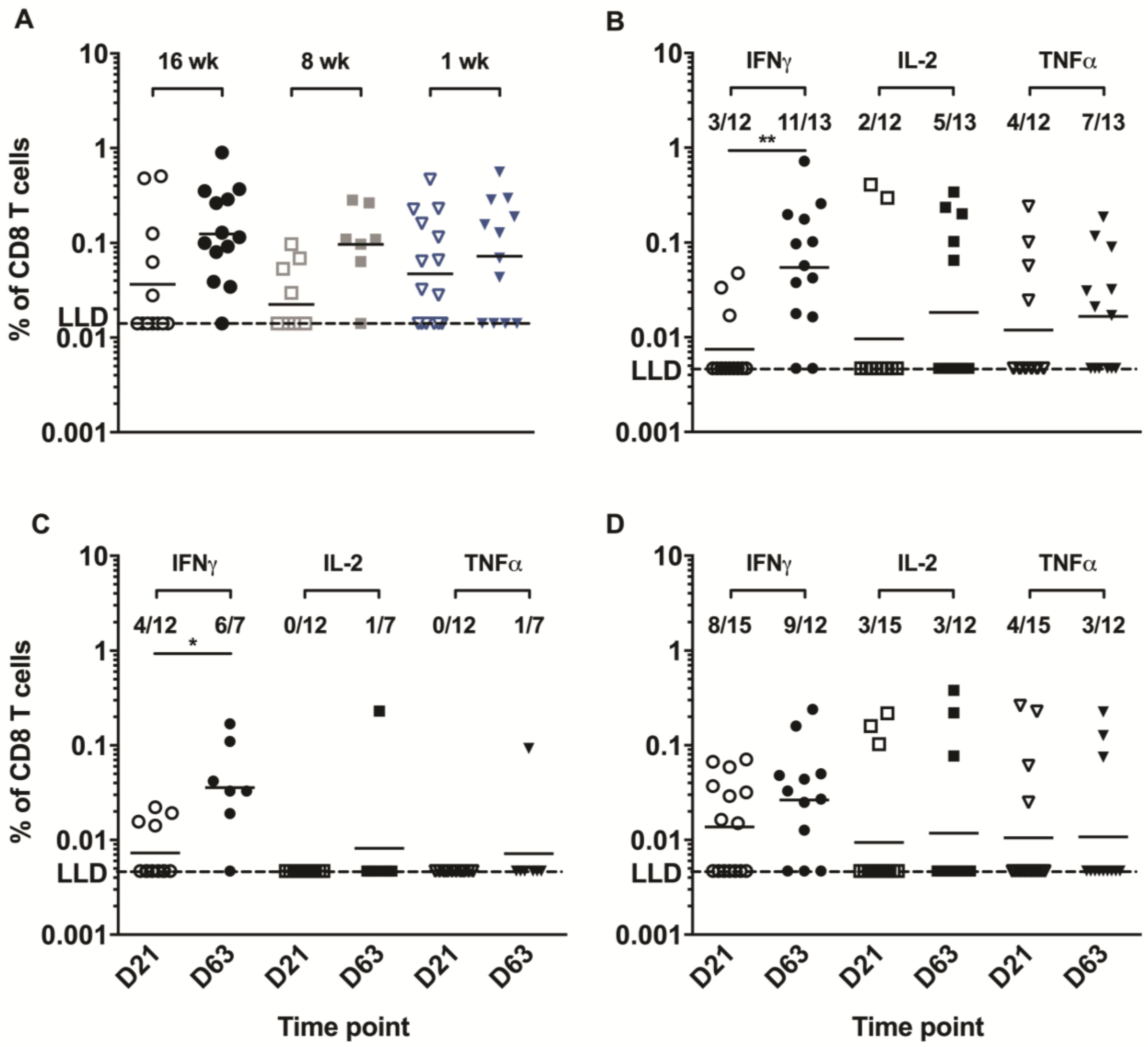

Supplementary Figure S5: Cytokine profile of TRAP-specific CD8$^+$ T cell response assessed by intracellular staining. Cytokine frequencies from CD8$^+$ T cells are shown 21 days post-prime with ChAd63 ME-TRAP (D21) and 7 days post-boost with MVA ME-TRAP (D63) in vaccinees of each age group. A - Total frequency of cytokine positive T cells; B - 16 week old; C - 8 week old; D - 1 week old group. Bars denote geometric mean. Numbers show the ratio of positive responses to total number of tested samples. LLD = lower limit of detection of the assay. Differences across groups in frequency of cytokine positive CD8$^+$ T cells and individual cytokine responses post-priming and post-boosting within each age group were analyzed using the Kruskal-Wallis test with Dunn's multiple comparison post-test, *p<0.05, **p<0.01.

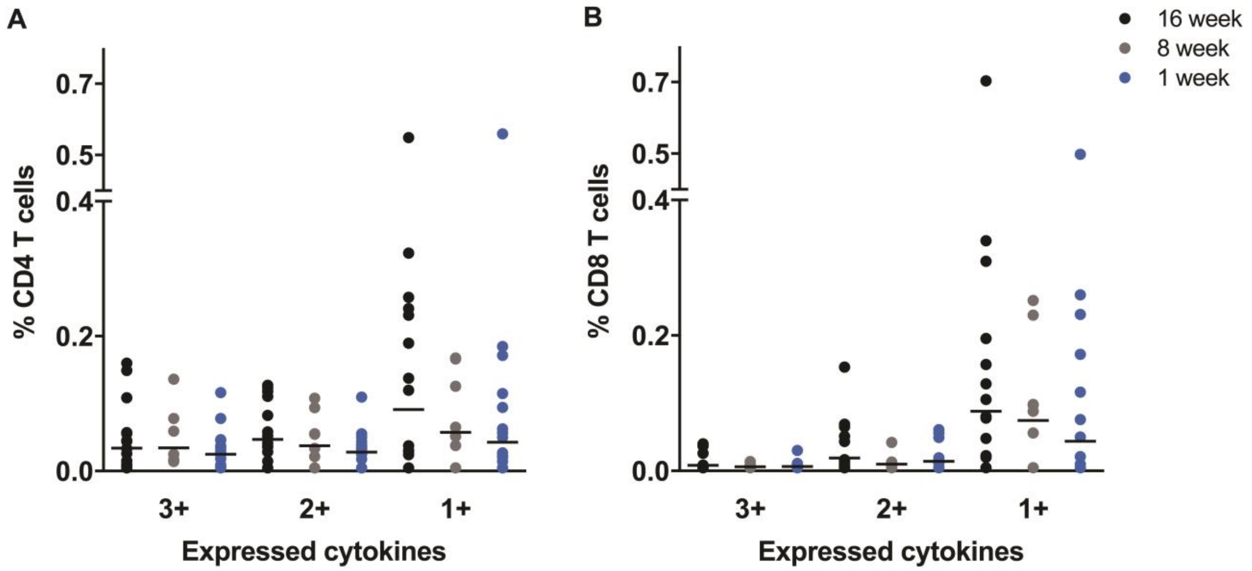

Supplementary Figure S6: Frequency of CD4+ and CD8+ T cells at day 63 producing 1 to 3 cytokines following *in vitro* recall with TRAP peptides pool. Scatter plots show individual peak responses to TRAP in each age group. T cell subsets were grouped according to the number of cytokines they simultaneously expressed (1, 2 or 3). Lines denote geometric mean percentages. Frequencies were compared between age group using the Kruskal-Wallis test with Dunn's multiple comparison post-test, *$p<0.05$.

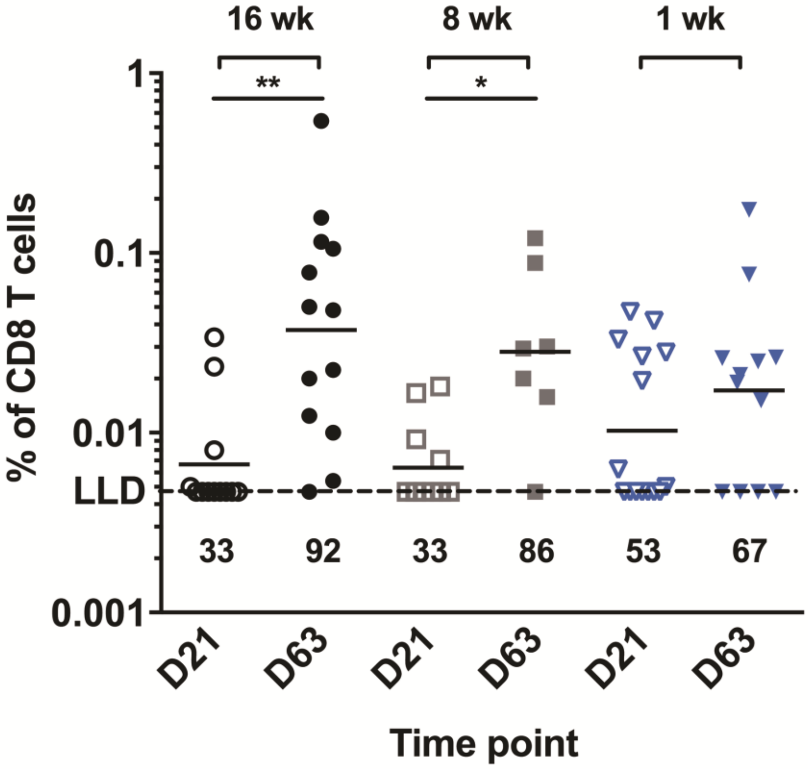

Supplementary Figure S7: Frequency of TRAP-specific monofunctional CD8^{+} T cells, producing IFNγ but negative for IL-2 and TNFα, in response to ChAd63 MVA ME-TRAP prime-boost immunization in Gambian infants. Scatter plot shows individual responses in each age group. Frequencies are shown 21 days after priming immunization with ChAd63 ME-TRAP (D21) and 7 days after boosting with MVA ME-TRAP (D63). Lines denote geometric mean percentages. Numbers in black represent the percentage of responders among the vaccinated infants for each time point. LLD = lower limit of detection of the assay. Differences in post-prime and post-boost frequencies within each age group and in peak responses between age group were compared using the Kruskal-Wallis test with Dunn's correction for multiple comparisons, *p<0.05, **p<0.01.

1.2 Supplementary Tables

Table S1: Schematic diagram showing IMP and EPI vaccinations across study groups

**Study visit**		**Screening**	**Day 0**	**Day 7**	**Day 21**	**Day 56**	**Day 63**	**Day 112**	**Day 168**	**Day 224**	**Day 252**
**Age at 1st vaccination**		**1 week***			**8 weeks**[†]		12 weeks	**16 weeks**[‡]	24 weeks		36 weeks
**IMP vaccination**			**Ad (prime)**			**MVA (boost)**					
**EPI vaccination**	**EPI dose**										
**BCG**	0.1ml	X									
**Hepatitis B**	0.5ml	X									
**Oral Polio**	2 drops	X			X		X	X			
**Pneumococcal Conjugate**	0.5ml				X		X	X			
**DTP Hib HepB**	0.5ml				X		X	X			
**Rotavirus**	1ml				X		X	X			
**Vitamin A**	1ml								X		
**Measles**	0.5ml										X
**Yellow fever**	0.5ml										X

Key:
[‡]Group 1 received first study vaccine at age of 16weeks
[†]Group 2 received first study vaccine at age of 8 weeks
*Group 3 received first study vaccine at age of 1 week
EPI-Expanded Programme on Immunization
IMP-Investigational Medicinal Product
BCG-Bacille Calmette Guerin
DTP-Diphtheria Tetanus Pertussis
Hib- Haemophilus Influenza B
Hep B- Hepatitis B
